# The research burden of randomized controlled trial participation: a systematic thematic synthesis of qualitative evidence

**DOI:** 10.1186/s12916-019-1476-5

**Published:** 2020-01-20

**Authors:** Nivantha Naidoo, Van Thu Nguyen, Philippe Ravaud, Bridget Young, Philippe Amiel, Daniel Schanté, Mike Clarke, Isabelle Boutron

**Affiliations:** 1 Université de Paris, CRESS, INSERM, INRA, F-75004 Paris, France; 2 Centre d’Epidémiologie Clinique, AP-HP, Hôpital Hôtel Dieu, F-75004 Paris, France; 3 Department of Health Services Research, Institute of Population Health Sciences, University of Liverpool, Liverpool, L69 3GB UK; 4 Department of Epidemiology, Mailman School of Public Health, Columbia University, New York, NY USA; 5 INSERM U 1123 ECEVE (Clinical Epidemiology, Economic, Evaluation, Vulnerable populations), University Paris Diderot, Paris, France; 6 Jusqu’A La Mort Accompagner la Vie (JALMALV), Strasbourg, France; 7 Comité des Utilisateurs et Professionnels, Institut National du Cancer (INCa), Paris, France; 8 Queen’s University Belfast School of Medicine Dentistry and Biomedical Sciences, Belfast, UK

**Keywords:** Randomized controlled trials, Research burden, Trial participation

## Abstract

**Background:**

Participation in randomized controlled trials (RCTs) may be quite demanding and could represent an important burden for patients. We aimed to explore this research burden (i.e., the psychological, physical, and financial burdens) experienced by patients through their participation in a RCT.

**Methods:**

We conducted a systematic review of qualitative studies exploring adult patients’ experiences with RCT participation. We searched MEDLINE (PubMed), CINAHL, PSYCHINFO, and Embase (search date March 2018) for eligible reports. Qualitative data coding and indexing were assisted by NVivo. The quality of reports was assessed by using the Critical Appraisal Skills Program (CASP) tool.

**Results:**

We included 45 qualitative studies that involved 1732 RCT participants. Important psychological burdens were identified at every stage of the trial process. Participants reported feeling anxiety and being afraid of “being a ‘guinea pig’” and described undergoing randomization and allocation to a placebo as particularly difficult resulting in disappointment, anger, and depression. Patients’ follow-up and trial closure were also responsible for a wide range of psychological, physical, and financial burdens. Furthermore, factors related to burdensome impacts and consequences were discerned. These factors involved trial information, poorly organized and too-demanding follow-up, and lack of appropriate management when the patient’s participation ended. Trial participation was also associated with beneficial effects such as the satisfaction of feeling “useful,” gaining “a sense of control,” and receiving special attention.

**Conclusions:**

Our finding provides a detailed description of research burden across the whole RCT process. Many of the burdens described could be anticipated, and some avoided in a movement toward minimally disruptive clinical research. Such an approach could improve trial recruitment and retention.

**Review registration:**

PROSPERO CRD42018098994

## Background

Randomized controlled trials (RCTs), and the systematic reviews that they contribute to, are considered the gold standard in clinical interventional research [1]. However, RCT participation may be quite challenging. Patients who are already trying to manage burdens associated with their illness and treatment could face additional burdens related to their trial participation. They may be required to travel, attend trial visits [2], undergo supplementary procedures (some of which may be invasive) [3], and complete trial questionnaires, among other demands that would not be necessary if they were not in a trial. All these tasks could be responsible for important psychological, physical, and financial burdens for patients, which may affect their willingness to begin and complete participation in a trial [4] and consequently have some implications for implementation science. However, despite hundreds of thousands of RCTs, relatively few studies have considered the burden of research participation on patients within the context of the trial itself.

Qualitative studies embedded or associated with RCTs could help understand patients’ experiences of RCT participation and the possible burdens encountered. To our knowledge, there are systematic reviews of qualitative studies to identify barriers and facilitators to recruitment and retention in RCTs [5–7]; however, no review yet has specifically explored patients’ research burden.

This study aimed to explore research burdens and benefits of adult patients’ participation in RCTs. This should allow the subsequent improvement of research planning and conduct.

In the context of this study, we defined “research burden” as encompassing the psychological, physical, and financial burdens to patients because of their participation in an RCT. We also defined patients as people with an illness.

## Methods

### Study design

We performed a systematic review and synthesis of qualitative research. We used thematic synthesis methods outlined by Thomas and Harden [8] and guidelines from the Cochrane Qualitative Methods Group [9]. We used an interpretative approach to go beyond the content of the original studies to develop analytical themes encompassing research burden. To ensure transparency, we adhered to the reporting guidelines set out by PRISMA [10] and ENTREQ (Additional file 1) [11].

### Search strategy

A preplanned search strategy was developed and structured around our research objective (Additional file 1) [12–16]. We searched MEDLINE (PubMed), CINAHL, PSYCHINFO, and Embase to identify qualitative studies including RCT participants to explore participants’ experiences. We also searched Google Scholar and retrieved all studies that cited each of 3 pre-specified “seed” publications [3, 17, 18]. All searches were performed in March 2018.

For each qualitative study, we systematically searched for the embedded or associated trial using the reference when available. When no data were available, we contacted the authors of the qualitative study.

### Screening

Inclusion criteria: We included all English-language reports of qualitative studies of patient participants of RCTs that explored their perspectives and experiences. We defined a qualitative study as having used or reported using qualitative methods for qualitative data collection and analysis. We also included studies using mixed methods if a clearly identifiable qualitative component was present. We defined an RCT as a study in which participants were randomly allocated to receive an intervention or a control.

Exclusion criteria: We excluded qualitative studies focusing on RCTs that included only children as well as RCTs that included only participants who had decreased decisional capacity or who required a proxy to consent to participate in the RCT. We also excluded qualitative studies of participants in screening trials, prevention trials, or only early-phase clinical trials as they may raise specific issues. Studies with mixed populations (e.g., patients and carers) were included, but only data related to patients were recorded. We also excluded studies including only potential participants or people who declined to join an RCT. Studies with both decliners and consenters were included, but only data related to consenters were recorded.

One review author (NN) independently screened all retrieved citations assisted by Covidence software. Full texts of eligible reports were retrieved and independently screened by one review author (NN).

### Data extraction

#### Quantitative data extraction

One review author (NN) extracted the following descriptive information from the qualitative reports using a standard data extraction form (Additional file 1): study design, data collection methods, data analysis methods, number of participants in the qualitative study, number of RCTs the participants were sourced from, whether the study was nested in the RCT, and which clinical domain(s) were involved.

Two review authors (NN, VNT) independently appraised the methodological quality [11, 19] by using the Critical Appraisal Skills Program (CASP) tool (Additional file 1), a widely used qualitative research appraisal checklist [20]. Disagreements were resolved by consensus and when needed, a third reviewer (IB). The weighted Cohen’s Kappa for agreement on CASP criteria ranged from 0.80 to 0.92.

We extracted the following information from the associated RCT reports by using a standard data extraction form (Additional file 1): clinical setting, geographical location, funding sources, number of trial arms, intervention, comparator, estimated sample size, number randomized, masking of allocation, and issues encountered.

#### Qualitative data extraction, analysis, and synthesis

We followed the detailed methods for thematic synthesis outlined by Thomas and Harden [8]. Each included full-text report underwent coding and analysis assisted by NVivo Pro v12. First, three independent review authors [NN, VNT, IB] inductively line-by-line coded a set of 10 reports. We pre-specified and coded the results/findings and discussion sections covering the authors’ interpretation of their data as well as any text reported as direct/verbatim patient quotes. Second, three reviewers (NN, VNT, IB) independently organized the open codes into structured descriptive themes based on similarities and differences between codes. Third, the three reviewers met to reach consensus on the codes and themes, with further interpretative discussion focused on the research question to generate analytical themes. Next, one reviewer (NN) independently coded the remaining reports, adding new excerpts to the pre-existing codes and themes in the codebook (Additional file 1) as well as creating new codes and themes as appropriate. Throughout the coding process, the review authors met regularly to cross-check newly generated codes and themes against the data, discuss interpretation, and synthesize analytical themes encompassing “research burden” and “research benefit.”

We presented examples of coded text excerpts in quoted italics followed by [PC] or [SC]. PC stands for “primary code,” i.e., the excerpt represents a verbatim patient quote, and SC stands for “secondary code,” i.e., the excerpt represents the author’s interpretation.

#### Patient and public involvement

The manuscript, codebook, figures, tables, and appendices were reviewed by a patient representative (DS), and helpful points further clarifying the wording of the interpretative analysis were adjusted for accordingly.

## Results

### Characteristics of qualitative studies

**Fig. 1:**
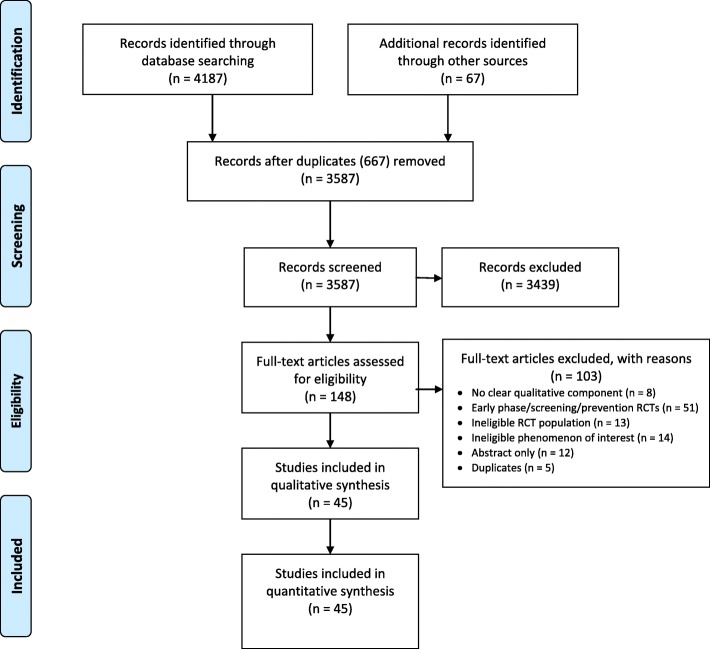
Systematic review flowchart

Of the 3587 records screened, we included 45 qualitative studies (Fig. 1; Additional file 1) involving 1732 patients who had participated in an RCT (Table 1). A qualitative design was used in 37 (82%) studies. The main methods used were interviews (*n* = 39; 87%) and thematic analysis (*n* = 21; 47%). The median number of participants in each study was 21 (Q1 to Q3: 15 to 38). The studies were mainly in the field of cancer (*n* = 14; 31%) and chronic diseases (*n* = 12; 27%). Six (13.3%) qualitative studies were published before 2005. Participants were sourced from a single RCT in 31 (71%) studies and from more than one RCT in 8 (16%). For 6 (13%) studies, the number of RCTs involved was unclear. The methodological quality is reported in Table 1. We were able to retrieve 42 RCTs: 37 were published final reports and 5 were published protocols (Table 2). Issues encountered during trial implementation were reported for 10 trials, 8 related to suboptimal recruitment such as slow accrual or high refusal rate and 2 to obtaining informed consent in an emergency setting.

**Table 1:** Characteristics of the primary qualitative reports

	*N* = 45 (100.0%)
Primary qualitative report characteristics	
Study design	
Qualitative	37 (82.2%)
Mixed methods	8 (17.8%)
Data collection methods	
Interviews	39 (86.6%)
Focus groups	3 (6.7%)
Surveys with open-ended questions	3 (6.7%)
Data analysis methods	
Content analysis	9 (20.0%)
Thematic analysis	21 (46.7%)
Grounded theory	11 (24.4%)
Interpretive phenomenological analysis	2 (4.4%)
Not reported	2 (4.4%)
For a single qualitative study, the RCT participants were sourced from:	
1 RCT**	31 (71.1%)
Multiple RCTs**	8 (15.5%)
Not reported***	6 (13.3%)
Number of RCT participants in each qualitative study	
Median (Q1, Q3); *n*	21.0 (15, 38); 1732
Was the primary qualitative study nested in the RCTs?	
Yes	27 (60.0%)
Clinical domain	
Oncology	14 (31.1%)
Chronic diseases	12 (26.7%)
Acute illnesses	4 (8.9%)
Mental health	1 (2.2%)
Trauma/orthopedics	2 (4.4%)
Obstetrics	8 (17.8%)
Urogynecology	2 (4.4%)
Mixed	2 (4.4%)
Publication year of primary qualitative study	
Before 2005	6 (13.3%)
CASP Tool Quality Appraisal	Yes
Q1 Was there a clear statement of the aims of the research?	45 (100.0%)
Q2 Is a qualitative methodology appropriate?	45 (100.0%)
Q3 Was the research design appropriate to address the aims of the research?	45 (100.0%)
Q4 Was the recruitment strategy appropriate to the aims of the research?	36 (80.0%)
Q5 Was the data collected in a way that addressed the research issue?	17 (37.8%)
Q6 Has the relationship between researcher and participants been adequately considered?	15 (33.3%)
Q7 Have ethical issues been taken into consideration?	42 (93.3%)
Q8 Was the data analysis sufficiently rigorous?	26 (57.8%)
Q9 Is there a clear statement of findings?	45 (100.0%)

*Due to rounding off numbers may not add up to 100

**31 qualitative studies involved a single RCT, and we were able to retrieve 27 RCT reports or protocols. 8 qualitative studies sourced participants from a total of 50 different RCTs, and we retrieved 15 RCT reports or protocols. Thus, a total of 42 RCT reports were available for description

***6 qualitative studies did not clearly report the number of RCTs that were involved

**Table 2:** Characteristics of the RCT reports from which primary qualitative studies sourced participants

	*N* = 42 (100.0%)
RCT characteristics	
Clinical setting	
Primary (home/GP/community based)	4 (9.5%)
Secondary (hospital based)	27 (64.3%)
Tertiary (specialized academic health facility based)	11 (26.2%)
Geographical location	
Europe	33 (78.6%)
America (USA and Central)	1 (2.4%)
Africa	3 (7.1%)
Asia	1 (2.4%)
Oceania	1 (2.4%)
Multi-continental	3 (7.1%)
RCT funding sources/sponsorship	
Non-profit/academic/public organizations	40 (95.0%)
Blinding of patients	
Yes	13 (31.0%)
Number of trial arms	
2	34 (81.0%)
3	7 (16.7%)
4	1 (2.4%)
Intervention **	
Drug (topical, oral, SC, IM, IV)	22 (52.4%)
Surgical procedure	4 (9.5%)
Participative (psychological, physical, educational, palliative, rehabilitative)	11 (26.2%)
Other	5 (11.9%)
Comparator**	
Placebo/Sham treatment	11 (26.2%)
Usual care/no treatment	18 (42.8%)
Active treatment	13 (31.0%)
Number of patients randomized	
Mean (SD); n***	1199 (1999); 46,748
Issues with RCT?	
None reported***	32 (76.2%)
Suboptimal recruitment (slow accrual, unwilling to be randomized, high refusal rate, regulatory delays, lack of eligible patients)	8 (19.0%)
Informed consent in an emergency	2 (4.8%)

*Due to rounding off numbers may not add up to 100

**If the RCT contained more than 2 arms, only 1 experimental intervention and 1 comparator was extracted

***There is missing data for 5 RCTs as only the protocol was available

### Burdensome impacts and consequences of trial participation

#### Psychological impacts

**Fig. 2:**
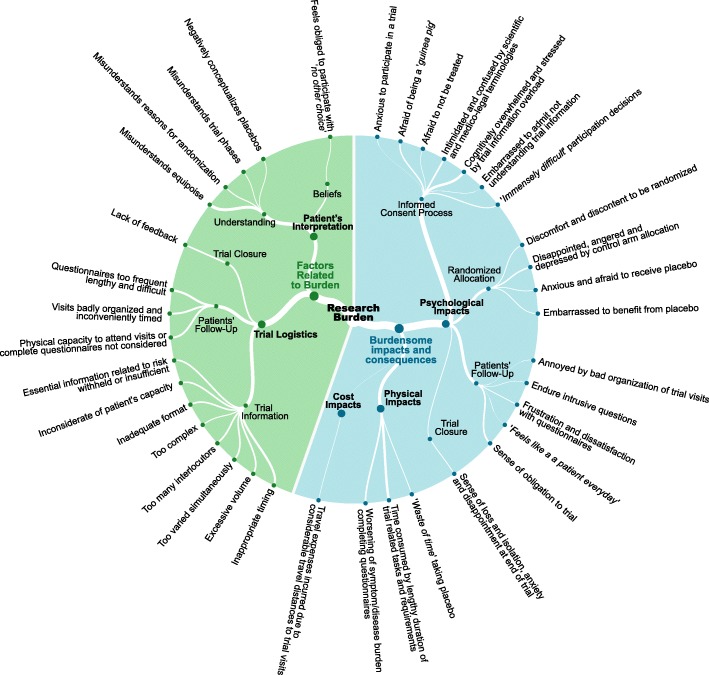
Classification of theme “Research Burden” and sub-themes “Burdensome Impacts and Consequences” and “Factors related to Burden”

The research burden experienced by participants in RCTs is detailed in Additional file 1 and summarized in Fig. 2. Participants endured a diverse array of psychological burdens at every stage of the trial process. The decision to commit to a trial, understand trial information, and provide consent was responsible for anxiety and fear of “being a ‘guinea pig’ and being used to “further the career of a scientist without benefit to one’s present condition” [SC]. Many participants felt stressed and overwhelmed when dealing with the “load of information” [PC], “intimidated” [SC] and confused by the complicated and technical terminologies used and embarrassed to admit not understanding trial information. Moreover, undergoing randomization carried significant psychological burden. Some struggled to accept the concept of random allocation: “deep inside, I believed the new treatment being better, and now I [agreed] to participate in a drawing of lots. I was not certain of winning the draw, if you understand? If I had got the standard treatment I maybe would have felt… it’s not as good as the one I’ve got. I think it’s wrong” [PC]. Patients expressed fear and anxiety associated with the possibility of placebo allocation: “Patients ‘run the risk’ of receiving the placebo (i.e., getting no treatment) and dying sooner” [SC]. Allocation to the control arm was associated with disappointment, anger and depression that was quite devastating: “…no hope for me… extremely depressed …went home and cried. To leave the hospital after an hour of filling forms … empty handed. I felt no one really understood how bad I felt” [PC].

Patients’ follow-up was also responsible for important psychological burdens. Frustration and dissatisfaction were associated with completing trial questionnaires: “Questionnaires are always terrible because you never can express by checking a box what one wants to say” [PC]. Finally, patients struggled to deal with the end of their participation in the trial and their transition to usual care. A sense of loss, isolation, and anxiety was associated with the end of the trial: “‘It was a bit of a blow.’; ‘in a way I suppose I was a bit anxious.’; ‘I felt as though I was losing friends here.’” [PC].

#### Physical impacts

Patients’ follow-up was also responsible for physical burdens. Particularly, “participants indicated they had experienced some negative effects from completing the outcome measures, chiefly that reading and rating their level of nausea and vomiting at a time when they were feeling nauseous had at times worsened their experience” [SC], which demonstrates direct physical burden. We also found that time consumed by trial participation was an important burden. One patient was “surprised by the fact that professionals presume that you have plenty of time” and further explained that “they did not take my possibilities and preferences into account” [PC]. Patients “had to travel to appointments in rush-hour traffic” [SC] and were forced to adapt their schedules to suit the trial. Patients reported allocation to the control arm or a placebo as a “waste of time” [PC], and there was a “personal belief [that patients] have little time left and their time would be shortened by participating in a clinical trial” [SC].

#### Cost impacts

It is also essential to consider the financial burden of trial participation: “the patient also lived a considerable distance from the hospital, and it seems he incurred considerable expense and inconvenience every time he attended an appointment” [SC].

#### Factors related to burden of trial participation

We further discerned factors related to research burden, which we differentiated into patients’ interpretation and trial logistics (Fig. 2; Additional file 1). Many factors were distinctly associated with trial information. Patients were overwhelmed due to inappropriate timing, excessive volume, inadequate format, and the variety and complexity of the information. Furthermore, some essential information related to participation risks was insufficient: “It would be good for people to be told up front how bad the chemo is. How incredibly tired you feel. That can’t be said enough.” [PC]. Factors related to beliefs and understanding featured prominently as a compounding issue such as “little or no understanding or consideration of the unknown and potentially equal risks and benefits of participating in the trial, and the principle of equipoise” [SC] induced or worsened burdensome impacts. Other important RCT concepts (such as reasons for using randomization) were similarly misunderstood by patients, contributing to discomfort and discontent related to being randomized. We also identified that factors related to patients’ follow-up, for example, frequency and length of trial questionnaires, worsened the additional workload: “It [questionnaire] was hard work because there were so many pages” [PC] and “I would have favored a weekly diary rather than a daily one” [PC]. Furthermore, attending poorly organized and inconveniently timed trial visits led to irritation: “The things that have annoyed me so far is you go to the [name of principal hospital in city 3], you’ve got to be there for eight o’clock in the morning, [and] they got no bed for you. So they can’t do anything until they have found a bed for you, and I’ve been there from eight until half past one, um, just hanging around” [PC].

Finally, disappointment at trial closure was punctuated by a lack of feedback related to results or allocation.

#### Beneficial effects of trial participation

Trial participation was also associated with beneficial effects (Table 3; Additional file 1). Patients believed their altruistic motivations and intentions to participate as being a “feel-good factor associated with their participation in a trial. Patients remarked that participation “makes you feel useful and gives satisfaction to take part in a trial [SC/PC]” and “it makes you feel better, doesn’t it, if you feel you are contributing something” [PC]. We also noted an emphasis on the relevance of patients’ participation for future research and the next generation of patients as “worthwhile” and appreciation for the opportunity to contribute meaningfully. Benefits were also reported, such as a newfound personal insight from completing trial questionnaires: “It gave me food for thought, it gave me more insight into perhaps what was really bothering me...some of the questions would bring to light, maybe some of the things I had been feeling, but didn’t realize it until I had to answer.” [PC] We also found that a sense of control may be regained: “And actually it helped because it was something positive to do, on certain days and ticking the boxes and all that sort of thing. I felt because I think part of having cancer is you lose control, and I am quite, the sort of person that likes to be in control and this is enabling me a little bit of control back” [PC]. Notably, an improved relationship with the trial staff and healthcare providers was identified, with trust, support, and encouragement being important components. Indeed, some patients described the trial clinic as being “like home from home [PC]” and a “safe haven or a pseudo-surrogate family” [PC]. Furthermore, receiving special attention such as closer supervision of illness and contact with healthcare staff, extra efforts, and superior facilities was considered as greatly advantageous. One impressed patient “felt privileged to be on a trial. We had a separate chemo facility and we all knew we were at the Ritz” [PC].

**Table 3:** Theme “Trial Participation Benefits”

Subthemes and codes	Primary text report excerpt
➢ Altruistic benefits	
• It feels good to do good	“This enabled women to continue to feel good about having taken part; they experienced the warm glow of having helped others”
• Contribution to future research	“If it can make it easier for somebody in the future, count me in”
• To ‘pay it forward’ and reciprocate previous generations’ contributions	“And I also looked at it like this: these are studies for the future, and after all I have a daughter and you never know. In that case I’m the kind of person to take part in things for other people, so that it’s better in the future than it is now, for example. What other people have done in the past, I’m making use of now”
• A way to give back to the health care service	“Undoubtedly the main motivational factor influencing participants was a desire to ‘give something back’”
➢ Personal benefits	
• Regain a sense of control	“Specifically, that it provided them with some control, at a time when most felt a lack of control over their cancer experience”
• Improve self-discipline	“Also, being enrolled in trials helped several participants to maintain self-discipline, crucial for people with chronic diseases who need to take drugs continuously”
• Less responsibility and workload	“This, combined with their fears of developing complications, had led them to value the input of UKPDS professionals who could ‘do the thinking, planning and worrying for [them]”
• Gain research knowledge	“All former trial participants said they felt more knowledgeable about trials and research since participating in a clinical trial”
• Increased health status awareness	“It also made me aware of any little changes....the answer might be, well, maybe a little different this time, or changed, which made me more aware of myself”
• Experience improved healthcare relationships	“Compassion, social support and communication related to development of positive and trusting relationships with the research team”
• Receive special attention	“felt privileged to be on a trial. We had a separate chemo facility and we all knew we were at the Ritz”
• Means to be gainfully occupied	“Yeah, yeah, I mean, it’s something to do, you know, it’s good fun, it breaks things up. Life gets a bit boring when you are stuck like this, you know”
• Monetary incentives	“However, the practical advantage to receive drugs immediately without pharmacy fees was appreciated. ‘Firstly the drugs were free, which I found good. And you did not have to pay five Euros [pharmacy charge], which was also a factor’”

## Discussion

To our knowledge, this is the first study providing a detailed description of the burdensome impacts experienced by participants in clinical trials and identification of factors associated with this burden.

This systematic review of 45 qualitative studies that involved 1732 RCT participants in various fields provides a detailed description of the diverse psychological, physical, and financial burdens experienced by patients when they participate in a trial. Burden arises across all stages of the trial: the informed consent process, randomization and masking, follow-up, and at the end of study participation. We further discerned factors associated with trial implementation as well as factors related to patients’ interpretation of trial concepts that are related to research burdens.

Research burden was initially conceptualized in the field of survey research with respondent burden, depending on the amount of effort required by the respondent, the amount of stress, and the length and frequency of interviews. This concept has progressively been extended to other types of research, with a particular focus on seriously ill patients [18]. Lingler and colleagues proposed a broader conceptualization of research burden and suggested the term “perceived participant burden” [21]. Some evidence shows that research burden can be common in RCTs. For example, half the respondents in an international survey of 2194 clinical trial participants considered trial participation as disruptive to their daily routine [22]. The concept of research burden has featured prominently in recent priority-setting partnerships for trial methodology research [23, 24].

### Implications

#### Impact on trial recruitment and retention

The high burden of trial participation could be an important barrier to trial participation and increase trial withdrawal. For instance, a study showed that the complexity and stringency of the protocol were among the most common reasons cited by patients as barriers to participation in clinical trials of cancer treatments [25]. Additional procedures and appointments, as well as travel time and travel costs, are frequent reasons for declining participation, missing appointments, and “withdrawing” from a trial [26]. These sentiments are an important issue because the success of RCTs depends on their ability to recruit a sufficient number of patients and to ensure that as few participants as possible withdraw from the research or become lost to follow-up. Yet, trial recruitment is generally slower than expected [27]. Many trials fail to reach their planned sample size within the timescale and funding originally envisaged. For example, less than one third of the trials from two UK academic funding institutions recruited 100% of their original target and 45% failed to recruit even 80% of the target sample size [28]. Similarly, a study of RCTs in the USA found that about one third of the trials recruited less than 75% of their planned sample [29]. The number of eligible participants declining participation varies according to the context of the study and can represent up to 50% of the patients invited to participate [30, 31]. Our results also have some implications for implementation science. Indeed, successful intervention implementation requires that patients accept trial participation. Slow recruitment increases cost and delays the transposition of results into practice. Results are less reliable if the planned sample size is not achieved [32, 33], and a high refusal rate raises important concerns about the external validity. The extrapolation of the findings to the target population will not be guaranteed [34–37], and the financial investment in the research might be wasted. This situation raises doubts about the extrapolation of trial findings to the future, target population [28, 34–37].

#### Ethical considerations

Further, there are important moral and ethical reasons for seeking to avoid unduly burdening patients. The process of seeking informed consent can, for example, be responsible for burden participants psychologically; it has even been considered cruel [38]. It can be very stressful for patients to listen to a physician describing the potential benefits of the new treatment and then be informed that the treatment will be decided by randomization. Nevertheless, research burden is probably insufficiently considered by regulatory agencies, which mainly focus on the direct risk induced by the interventions and data collection. This burden is also inadequately considered by researchers who may be too busy focusing on the comprehensiveness, quality, and appropriate standardization of the data collected.

#### Toward minimally disruptive and compassionate clinical research

Funders, trialists, and methodologists should rethink the planning and conduct of trials. First, they must minimize research burdens and should implement “minimally disruptive and compassionate clinical research [39]”. Second, in the same way, a framework has been developed for the humanization of healthcare [40]; we need to move toward compassionate clinical research. Indeed, our results highlight that most of the burden experienced by participants are psychological burdens. While it is impossible to completely reduce this burden, trialists could implement interventions to favor compassionate and empathic support to patients. Such support has demonstrated its beneficial effect for healthcare [41]. The results of this review could help improve the design and conduct of RCTs by helping researchers to identify and implements strategies to reduce research burden. Such strategies might be implemented and evaluated in a Study Within A Trial (SWAT) [42] and thereby add to the evidence base on recruitment and retention in trials [5, 6]. Further, we need to explore other study designs that could reduce burden and improve external validity such as trials embedded in registries, cohorts, or routinely collected data [43] but also observational studies and use of routinely collected data. Patient and public involvement at the planning stage of the trial could also help reduce the burden [44–46] and drawing on patients’ experiential knowledge, ideas, and input when designing interventions to reduce research burden is essential [47–50]. The amount of data recorded as well as the modalities for recording data could also be questioned. For instance, O’Leary and colleagues showed that the median number of items collected per participant in a sample of cancer RCTs was 599 (range 186 to 1035), but only 18% of the data collected was actually used and reported in the resulting articles [46]. Similarly, a retrospective study of patient travel burden in cancer clinical trials using a Google Maps calculator showed that the median unidirectional distance traveled from home to the study site was 40 km (interquartile range 17.7 to 120.7) [51]. The benefits of trial participation identified in this study should also be considered for the planning and conducting of future research although it is important to acknowledge that some factors (e.g., completion of questionnaire) were considered as beneficial for some patients and burdensome by others.

### Limitations

There are limitations to our study. First, although our search strategy aimed to be comprehensive, we cannot exclude that we may have missed some studies. Nevertheless, the guidance for qualitative reviews focuses more on confidence in the synthesized findings, rather than on comprehensiveness [52]. Second, privacy and confidentiality reasons meant that we did not contact the authors of the included studies to request their complete data, and therefore, our thematic analysis and synthesis was limited to the findings and patient quotes published in the qualitative reports. Further, for feasibility reasons, we excluded some types of patients from this research burden project, such as adults with decreased decisional capacity for consent or requiring a proxy and children. We did not consider decliners although they have a potentially valuable perspective on research burden. We also excluded early-phase trials as well as screening trials or prevention trials.

Finally, we could not take into account participants’ individual health status, i.e., disease progression and treatment burdens, as well as their personal capacity which could highly impact the burden experienced.

## Conclusion

Our findings provide a detailed description of research burden across the whole RCT process. This compendious representation further elucidates key factors related to burden. The consideration of these modifiable factors in the planning and design of RCTs, such as the timing, volume, complexity, or format of trial information or the organization of patients’ follow-up, could help perform better RCTs. Many of the burdensome impacts described could be anticipated, and some avoided in a movement toward minimally disruptive clinical research. Such approach could improve trial recruitment and retention.

## Supplementary information

**Additional file 1.** ENTREQ checklist. Search strategies. Data extraction form. CASP tool. Codebook. Table of primary full text reports included in systematic review and thematic synthesis.

## Data Availability

Dataset available from the corresponding author (isabelle.boutron@aphp.fr).

## Abbreviations

CASP: Critical Appraisal Skills Program
PC: Primary code
RCT: Randomized controlled trial
SC: Secondary code
