# Supplementary material for: The research burden of randomized controlled trial participation: a systematic thematic synthesis of qualitative evidence
Source: BMC Med. 2020 Jan 20;18:6. doi: 10.1186/s12916-019-1476-5 (PMC6970283; doi:10.1186/s12916-019-1476-5)
Supplement: Supplementary file 1 — Additional file 1. ENTREQ checklist. Search strategies. Data extraction form. CASP tool. Codebook. Table of primary full text reports included in systematic review and thematic synthesis. [file 12916_2019_1476_MOESM1_ESM.docx]

**Additional file 1**

1. ENTREQ checklist
2. Search strategies
3. Data extraction form
4. CASP tool
5. Codebook
6. Table of primary full text reports included in systematic review and thematic synthesis

1. **ENTREQ (Enhancing Transparency in reporting the synthesis of qualitative research) Checklist**

| **No** | **Item** | **Guide and description** | **Comment where reported** |
| --- | --- | --- | --- |
| 1 | Aim | State the research question the synthesis addresses. | See Abstract and Background |
| 2 | Synthesis methodology | Identify the synthesis methodology or theoretical framework which underpins the synthesis, and describe the rationale for choice of methodology (e.g. meta-ethnography, thematic synthesis, critical interpretive synthesis, grounded theory synthesis, realist synthesis, meta-aggregation, meta-study, framework synthesis). | See Title, Abstract and Methods: Study Design |
| 3 | Approach to searching | Indicate whether the search was pre-planned (comprehensive search strategies to seek all available studies) or iterative (to seek all available concepts until they theoretical saturation is achieved). | See Methods-Search strategy |
| 4 | Inclusion criteria | Specify the inclusion/exclusion criteria (e.g. in terms of population, language, year limits, type of publication, study type). | See Methods- Screening |
| 5 | Data sources | Describe the information sources used (e.g. electronic databases (MEDLINE, EMBASE, CINAHL, psycINFO, Econlit), grey literature databases (digital thesis, policy reports), relevant organisational websites, experts, information specialists, generic web searches (Google Scholar) hand searching, reference lists) and when the searches conducted; provide the rationale for using the data sources. | See Methods-Search Strategy |
| 6 | Electronic search strategy | Describe the literature search (e.g. provide electronic search strategies with population terms, clinical or health topic terms, experiential or social phenomena related terms, filters for qualitative research, and search limits). | See Methods-Search Strategy and Appendix 1 |
| 7 | Study screening methods | Describe the process of study screening and sifting (e.g. title, abstract and full text review, number of independent reviewers who screened studies). | See Methods- Screening |
| 8 | Study characteristics | Present the characteristics of the included studies (e.g. year of publication, country, population, number of participants, data collection, methodology, analysis, research questions). | See Table 1 and Appendix 3 |
| 9 | Study selection results | Identify the number of studies screened and provide reasons for study exclusion (e,g, for comprehensive searching, provide numbers of studies screened and reasons for exclusion indicated in a figure/flowchart; for iterative searching describe reasons for study exclusion and inclusion based on modifications t the research question and/or contribution to theory development). | See Figure 1 Prisma Flowchart |
| 10 | Rationale for appraisal | Describe the rationale and approach used to appraise the included studies or selected findings (e.g. assessment of conduct (validity and robustness), assessment of reporting (transparency), assessment of content and utility of the findings). | See Appendix 2 |
| 11 | Appraisal items | State the tools, frameworks and criteria used to appraise the studies or selected findings (e.g. Existing tools: CASP, QARI, COREQ, Mays and Pope[[25](https://bmcmedresmethodol.biomedcentral.com/articles/10.1186/1471-2288-12-181#CR25)]; reviewer developed tools; describe the domains assessed: research team, study design, data analysis and interpretations, reporting). | See Methods-Quality Appraisal and Appendix 2 |
| 12 | Appraisal process | Indicate whether the appraisal was conducted independently by more than one reviewer and if consensus was required. | See Methods-Quality Appraisal |
| 13 | Appraisal results | Present results of the quality assessment and indicate which articles, if any, were weighted/excluded based on the assessment and give the rationale. | See Results-Description of quantitative data and quality appraisal and Table 1 |
| 14 | Data extraction | Indicate which sections of the primary studies were analysed and how were the data extracted from the primary studies? (e.g. all text under the headings “results /conclusions” were extracted electronically and entered into a computer software). | See Methods- Qualitative Data Extraction, Analysis and Synthesis |
| 15 | Software | State the computer software used, if any. | NVIVO |
| 16 | Number of reviewers | Identify who was involved in coding and analysis. | 3 review authors (NN) (VNT) (IB) |
| 17 | Coding | Describe the process for coding of data (e.g. line by line coding to search for concepts). | See Methods- Qualitative Data Extraction, Analysis and Synthesis |
| 18 | Study comparison | Describe how were comparisons made within and across studies (e.g. subsequent studies were coded into pre-existing concepts, and new concepts were created when deemed necessary). | See Methods- Qualitative Data Extraction, Analysis and Synthesis |
| 19 | Derivation of themes | Explain whether the process of deriving the themes or constructs was inductive or deductive. | See Methods- Qualitative Data Extraction, Analysis and Synthesis |
| 20 | Quotations | Provide quotations from the primary studies to illustrate themes/constructs, and identify whether the quotations were participant quotations of the author’s interpretation. | See Results-Thematic analysis and synthesis |
| 21 | Synthesis output | Present rich, compelling and useful results that go beyond a summary of the primary studies (e.g. new interpretation, models of evidence, conceptual models, analytical framework, development of a new theory or construct). | See Results-Thematic analysis and synthesis |

1. **Search strategies**

| **Search strategies** | |
| --- | --- |
| **Database and Search date** | **Search terms** |
| Pubmed (MEDLINE)  24 March 2018 | (“trial participants” OR “trial participation”) AND (“experiences” OR “perceptions” OR “attitudes” OR “beliefs” OR “opinions” OR “views” OR “burdens”) Limiters-Abstract available. Narrowed by Language: English Sorted by best match. |
| CINAHL  24 March 2018 | (“trial participants” OR “trial participation”) AND (“experiences” OR “perceptions” OR “attitudes” OR “beliefs” OR “opinions” OR “views” OR “burdens”) Limiters-Abstract available. Narrowed by Language: English |
| PsychINFO  24 March 2018 | (“trial participants” OR “trial participation”) AND (“experiences” OR “perceptions” OR “attitudes” OR “beliefs” OR “opinions” OR “views” OR “burdens” OR “barriers”) Limiters-Abstract available. Narrowed by Language: English |
| Embase  23 March 2018 | (“trial participants” OR “trial participation”) AND (“experiences” OR “perceptions” OR “attitudes” OR “beliefs” OR “opinions” OR “views” OR “burdens” OR “barriers”) Limiters-Abstract available. Narrowed by Language: English |

1. **Data extraction form**

| Quantitative Data |  |
| --- | --- |
| Primary qualitative study characteristics |  |
|  | Study design:  0=Qualitative design  1=Mixed methods design |
|  | Year of publication of primary qualitative report |
|  | Primary study data collection methods  0=Interviews  1=Focus groups  2=Surveys with open ended questions  3=Other  4=NR |
|  | Number of participants in primary qualitative study:  n=x |
|  | Primary qualitative study data analysis methods  0=Content analysis (can be conventional direct or summative)  1=Thematic analysis  2=Grounded theory  3=Interpretive phenomenological analysis  4=Other  5=NR |
|  | Funding sources for the primary qualitative study?  0=Profit  1=Non-profit  2=NR  3=No funding |
|  | Is the primary qualitative study nested within a single RCT?  0=Yes  1=No |
| RCT characteristics |  |
|  | Number of RCTs in primary qualitative study:  0=NR  1=1,  n=X |
|  | RCT setting  0=Primary care setting  1=Secondary care settings  2=Tertiary care setting  3=Other  4=NR |
|  | RCT location continent  0=Europe  1=America (North and US)  2=South America  3=Africa  4=Asia  5=Oceania  6=Multinational  7=NR |
|  | Number of patients randomized: 0=NR  n=X |
|  | Number of trial arms:  0=NR  n=X |
|  | Intervention  0=Drug  1=Drug topical  2=Drug oral/SC/IM  3=Drug IV  4=Surgical  5=Participative Psychological  6=Participative Physical  7=Participative Educational 8=Participative Palliative/Rehabilitative  9=Other  10=NR |
|  | Comparator  0=Placebo/Sham treatment  1=Standard or Usual care/No treatment  2=Active treatment  3=Other  4=NR |
|  | Masking of allocation  0=Yes  1=No  2=NR |
|  | Clinical domain  0=Cancer (Ca)  1=Chronic diseases (Ch)  2=Acute illnesses (Ai)  3=Orthopedics (Or)  5=Obstetrics (Ob)  6=Uro-gynecology (Ug)  7=Mixed (Mi) |

1. **CASP tool**

2 review authors independently performed a quality appraisal of the included qualitative studies to assess the methods and whether the findings were grounded in the patient’s experiences of trial participation. Several points must be emphasized for the interpretation of the quality appraisal[[37](#_ENREF_37)]. First, each of the 10 criteria in the CASP tool may be assessed with either ‘Yes’, ‘No’ or ‘Unclear’ response and no specific weight is assigned per question. As such, there is no overall numerical score for quality per study. Further, the CASP tool has been criticized for a lack of sensitivity to interpretative validity (“The degree to which participants’ viewpoints, thoughts, intentions, and experiences are accurately understood and reported by the qualitative researcher”) and theoretical validity (“The degree to which a theory or theoretical explanation informing or developed from a research study fits the data and is, therefore, credible and defensible”)[[38](#_ENREF_38)]. Therefore, our appraisal strategy included the consideration of the additional bullet-points CASP supplied for each criteria(Appendix 3). If one bullet-point was not satisfied the report was downgraded to ‘Unclear.’ If two or more bullet-points were not satisfied, the report was downgraded to ‘No’. The missing bullet-points were elicited with the assessment for each criterion.

| **CRITICAL SKILLS APPRAISAL PROGRAMME (CASP) TOOL** |
| --- |
| **Section A: Are the results valid?** |
|  |
| 1. Was there a clear statement of the aims of the research? HINT- Consider: |
| • what was the goal of the research |
| • why it was thought important |
| • its relevance |
|  |
| 1. Is a qualitative methodology appropriate? HINT- Consider: |
| • If the research seeks to interpret or illuminate the actions and/or subjective experiences of research participants |
| • Is qualitative research the right methodology for addressing the research goal |
|  |
| **Is it worth continuing?** |
|  |
| 1. Was the research design appropriate to address the aims of the research? HINT- Consider: |
| • if the researcher has justified the research design (e.g. have they discussed how they decided which method to use) |
|  |
| 1. Was the recruitment strategy appropriate to the aims of the research? HINT- Consider: |
| • If the researcher has explained how the participants were selected |
| • If they explained why the participants they selected were the most appropriate to provide access to the type of knowledge sought by the study |
| • If there are any discussions around recruitment (e.g. why some people chose not to take part) |
|  |
| 1. Was the data collected in a way that addressed the research issue? HINT- Consider: |
| • If the setting for the data collection was justified |
| • If it is clear how data were collected (e.g. focus group, semi-structured interview etc.) |
| • If the researcher has justified the methods chosen |
| • If the researcher has made the methods explicit (e.g. for interview method, is there an indication of how interviews are conducted, or did they use a topic guide) |
| • If methods were modified during the study. If so, has the researcher explained how and why |
| • If the form of data is clear (e.g. tape recordings, video material, notes etc.) |
| • If the researcher has discussed saturation of data |
|  |
| 1. Has the relationship between researcher and participants been adequately considered? HINT- Consider: |
| • If the researcher critically examined their own role, potential bias and influence during (a) formulation of the research questions (b) data collection, including sample recruitment and choice of location |
| • How the researcher responded to events during the study and whether they considered the implications of any changes in the research design |
|  |
| **Section B: What are the results?** |
|  |
| 1. Have ethical issues been taken into consideration? HINT- Consider: |
| • If there are sufficient details of how the research was explained to participants for the reader to assess whether ethical standards were maintained |
| • If the researcher has discussed issues raised by the study (e.g. issues around informed consent or confidentiality or how they have handled the effects of the study on the participants during and after the study) |
| • If approval has been sought from the ethics committee |
|  |
| 1. Was the data analysis sufficiently rigorous? HINT- Consider: |
| • If there is an in-depth description of the analysis process |
| • If thematic analysis is used. If so, is it clear how the categories/themes were derived from the data |
| • Whether the researcher explains how the data presented were selected from the original sample to demonstrate the analysis process |
| • If sufficient data are presented to support the findings |
| • To what extent contradictory data are taken into account |
| • Whether the researcher critically examined their own role, potential bias and influence during analysis and selection of data for presentation |
|  |
| 1. Is there a clear statement of findings? HINT- Consider: |
| • If the findings are explicit |
| • If there is adequate discussion of the evidence both for and against the researcher’s arguments |
| • If the researcher has discussed the credibility of their findings (e.g. triangulation, respondent validation, more than one analyst) |
| • If the findings are discussed in relation to the original research question |
| Section C: Will the results help locally? |
|  |
| 1. How valuable is the research? HINT- Consider: |
| • If the researcher discusses the contribution the study makes to existing knowledge or understanding (e.g. do they consider the findings in relation to current practice or policy, or relevant research based literature |
| • If they identify new areas where research is necessary |
| • If the researchers have discussed whether or how the findings can be transferred to other populations or considered other ways the research may be used |

1. **Codebook**

| **CODEBOOK ALL INCLUDED TEXTS (n=45)**  *Cancer = Purple n=14*  *Chronic Diseases = Blue n=12*  *Acute Illnesses = Green n=4*  *Mental Health = Red n=1*  *Ortho = Pink n=2*  *Obstetrics = Orange n=8*  *UroGynae = Yellow n=2*  *Mixed = Black n=2* | | |
| --- | --- | --- |
| **Themes , Subthemes and Codes (Total number of source texts)** | **Definitions** | ***Text excerpt example with source report number and whether primary or secondary code*** |
| **THEME: Research Burden** | **The burden experienced by patients through trial participation** | |
| **SUBTHEME: Factors Related to Burden (28)** | **Factors that may induce or worsen burdens** | |
| Patient’s Interpretation(12) | Factors related to the patient | |
| - Understanding (12) | Patient’s level of research literacy is insufficient and understanding of trial concepts and process is limited | |
| - - Misunderstands Equipoise(10) |  | |
| - - - Therapeutic Misconception(8) | *1SC “As such, there was sometimes little or no understanding or consideration of the unknown and potentially equal risks and benefits of participating in the trial, and the principle of equipoise.”*  *1SC “This motivation may have influenced a cognitive bias towards the potential benefit of treatment, which seemed to dominate much of the talk about the trial amongst intervention participants, several of whom emphasized potential anti-cancer, curative effects of the treatment.”*  *1SC “Amongst the majority of patients, there was also little consideration of the unknown and potentially equal risks and benefits of participating in the trial.”*  *1SC “These beliefs provided the basis for intervention participants’ acceptance and adherence to their injection regime and could be considered indicative of ‘therapeutic misconception’”*  *1SC “The focus on treatment benefit was similarly evident in the ways in which intervention participants felt about their injection regime. Although some participants did not like injections, and most participants experienced mild discomfort and pain as well as bruising around the site of the injection, they tolerated daily injections because of the potential benefits that they might get from treatment.”*  *1SC “In the later interviews, participants continued to tolerate injecting, despite continuing discomfort, on the grounds that it is ‘doing me good’.”*  *2SC “The majority of established patients who had previously participated in a treatment trial were unable to distinguish between making a decision to come to the cancer center to seek treatment and agreeing to a clinical trial.”*  *11SC “The most commonly perceived personal benefit was to receive more effective treatment from the clinical trial than what is currently available, despite having been informed that the treatment is still experimental”*  *11SC “Most participants (24/29) believed that the benefit from the clinical trial was mostly for them personally.”*  *19SC “therapeutic misconception and in particular, therapeutic mis-estimation emerged as an influencing factor on how a number of participants described their decision-making and expected personal outcomes in relation to HSCT and ASTIC study participation.”*  *30PC “I understand that I will get treatment and I will recover, by the grace of Allah. They told me many things but I didn’t understand everything.”*  *30SC “They considered the clinical study aimed at providing good treatment and individual cure from the illness”*  *38SC ‘There was limited appreciation of any potential for harm to either the woman or her unborn baby; and a clear belief among the women that magnesium sulphate was beneﬁcial.’*  *51SC “A small number of people indicated that they would have preferred to receive the active treatment as opposed to the placebo”*  *APC “I would be very disappointed if he would end up in the control group.”* | |
| - - - Perception that the Trial Provides Individual Therapeutic Benefit(3) | *11SC “The most commonly perceived personal benefit was to receive more effective treatment from the clinical trial than what is currently available, despite having been informed that the treatment is still experimental”*  *11SC “Most participants (24/29) believed that the benefit from the clinical trial was mostly for them personally.”*  *19SC “therapeutic misconception and in particular, therapeutic mis-estimation emerged as an influencing factor on how a number of participants described their decision-making and expected personal outcomes in relation to HSCT and ASTIC study participation.”*  *30PC “I understand that I will get treatment and I will recover, by the grace ofAllah. They told me many things but I didn’t understand everything.”*  *30SC “They considered the clinical study aimed at providing good treatment and individual cure from the illness”* | |
| - - - Unrealistic Optimism(3) | *1PC “But apparently it could help shrink the cancers as well…That’s what they are hoping for.”*  *19SC “For others, expectations were greater, and the trial was described as offering a potential cure.”*  *19PC “Definitely, yeah. I mean, when I first heard about it, I thought it was going to be this like, cure”*  *30SC “Others mentioned the purpose of hospital admission was to ensure cure from the illness.”*  *30SC “They considered the clinical study aimed at providing good treatment and individual cure from the illness”* | |
| - - - Allocation by Randomization is Unethical and “Wrong”(2) | *10SC “A general discomfort with the randomisation in itself was articulated across interviews. Most patients, however, accepted the procedure, although many did not understand the reason for it.”*  *10PC “…They spoke highly about that chemo I got. However, they can just give it to me. Why must I be put in a pool, [of patients] and then toss a coin about it? … I just think that the drawing of lots is strange. Why can’t they just give you the one they think is the most effective? I did not understand … [patient 2 (breast cancer, trial participant, CEF)]”*  *10SC “Yet, the major problem for most patients in relation to randomisation was that they did not get the impression of true equipoise between treatments. Practically all the trial participants wanted to get the new experimental treatment, irrespective of possible graver adverse effects. They strongly expressed unease and discontent with not just ‘‘getting it’’ and having to accept a drawing of lots (ie, randomisation), and some of them even considered the procedure unethical due to the perceived lack of clinical equipoise.”*  *10PC “… deep inside I believed the new treatment being better, and now I accepted to participate in a drawing of lots. I was not certain of winning the draw, if you understand? If I had got the standard treatment I maybe would have felt… it’s not as good as the one I’ve got. I think its wrong …”*  *40SC “Randomization was perceived negative and resulted in uncertainty. Women could not explain (in any way) why randomization was used, or could be used. This seemed based on a combination of not knowing why randomization could or should be used, and not trusting that there is really equipoise and the doctor does not know what is best for them.”* | |
| - - Misunderstands Reasons for Randomization(3) | *1PC “Well, it’s someone who doesn’t know you doing it. So it’s not favouritism: ‘Oh yeah, that’s a friend of mine, put him on it, like so’; so, yeah, it’s cuddling up, you know. It’s just that nobody knows you; um, they make a decision on the facts that they are given, and that’s it.”*  *1SC “Other patients clearly misunderstood the selection process.”*  *1SC “As in previous studies, a number of participants had misunderstood the process of random selection [3–7] and in some cases perceived treatment allocation based on clinical assessment.”*  *1SC “Several patients thought that a medical decision had been made about which arm they should be in based on some kind of clinical assessment of their condition, in one case following the results of a blood test”*  *10SC “In addition, two patients believed that they had some influence in the choice of treatment despite clearly recognising the allocation of treatments by chance (patient 1 (breast cancer, trial participant, ovarian radiation) and patient 27 (ovarian cancer, trial participant, treated with paclitaxele/epirubicine/ carboplatine).”*  *10SC “A few patients thought that the randomisation was used as a way of rationing scarce resources.*  *18SC “while before finding out what treatment he had received, David thought that his practitioner would not give him placebo acupuncture because she cared for him and he trusted her.”* | |
| - - Misunderstands Trial Phases(2) | *2SC “The majority of participants said they did not know that trials were categorized in phases.”*  *11SC “Most participants had a poor understanding of a Phase 3 study”*  *11PC “Examples of participants’ responses that seemed to have a poor understanding are as follows:*  *• “I have no idea.” • “The fact that I’m now in phase 3 means that I am progressing well. The treatment is working.” • “In phase 3 there are more tests to check if the cancer has stopped or not.”* | |
| - - Negatively Conceptualizes Placebos(2) | *18SC “After finding out that he had been receiving placebo, Ben suggested that it might be the treatment that investigators give you if they don’t want you to get better”*  *18SC “while before finding out what treatment he had received, David thought that his practitioner would not give him placebo acupuncture because she cared for him and he trusted her.”*  *18SC/PC “When asked how she felt about not knowing which treatment she would receive, Emily revealed that she thought she would be able to tell which treatment she was receiving based on its effects. If she did not get any benefit from treatment then she would assume she was receiving placebo acupuncture: “I think I would know, because if there’s no improvement I know it wouldn’t be the acupuncture.”*  *18SC “Being ‘tricked’ into feeling better was seen as making a person appear gullible”*  *39SC “Two women believed that their part in the research was less valuable if they took the placebo and one thought that if she was taking the placebo she was not in the trial.”* | |
| - Personal Beliefs Related to Trials(4) | Patient’s personal beliefs related to clinical trials | |
| - - “Something is better than nothing”(3) | *1SC “Other participants in the intervention group felt obliged to try what was available to them on the grounds that it might work, rather than have nothing.”*  *1PC “Well, no, I’d rather have something that might prevent it rather than have nothing, you know. Half a defence is better than no defence. So, that’s what I thought, I thought, well, uh, well it mightn’t, but then again if it does.”*  *1SC “Several participants were willing to try anything that was suggested to help them, implying a degree of desperation and potential vulnerability, especially for the patient who reported having little understanding of how the treatment worked.”*  *19SC/PC “Participants described having ‘no other choice’”*  *38PC “Anything’s a bonus if it’s gonna help then we’ve got to try it. I was hoping and praying that it would help”* | |
| - - Perceives trial as last chance or final hope(2) | *2SC “The majority said they did not really know what a clinical trial was or felt that trials were a last chance for someone who has no hope; what one does when the current treatment is not working; an indication by the physician that death from cancer is imminent; for rare types of cancer or unknown disease.”*  *2SC “Also, one patient wondered whether she had been offered the trial because other options were not working.”*  *2SC “I always wondered if this was a “Hail Mary.” It wouldn’t have changed my decision to go on the trial, but would have been nice to know”*  *19SC/PC “for many that this treatment was a ‘last hope’.”* | |
| Trial Logistics(22) | Factors related to the trial processes such as the consent process and the follow up process | |
| - Trial Information(12) | Factors related to the timing, volume, content, format and manner of relaying or presenting trial information to patients | |
| - - Inappropriate Timing(7) | *1SC “One participant felt overloaded with information from a number of different health professionals at a difficult and emotional time”*  *1SC “Trial information was given at a time when patients had a lot of information to take in about their diagnosis and treatment.”*  *1PC “It was almost at the time I was just starting chemo, so I had a load of information from the lung nurse, from the doctor… the specialist, uh, which quite honestly was almost an overload.”*  *7SC/PC “In this cancer outpatient service, women were at different points in their identiﬁcation, adjustment and management of illness. Receiving new information about a diagnosis and/or options about cancer treatment left women to assimilate this information and their reactions to the details, while trying to participate effectively in care planning decisions. They felt bafﬂed with the amount of information and found it difﬁcult to concentrate during the consultation, picking up on only some bits of the information. ‘ … to tell you the truth I don’t know that day to this what the doctor, consultant I should call him, said to me because the only thing I picked up on was chemotherapy. “What you telling me?” and that was it. So … ’ (SN8, ovarian) Receiving an opportunity to take part in a trial at this point added to the complexity of this process, especially when the new treatments offered in the trial and standard treatments appeared similar, and impacted on how they reasoned about the different options. Women found it difﬁcult to differentiate between treatments offered as standard and within the trial.”*  *24SC “Some did feel that being in pain on arrival, feeling overwhelmed, alone or anxious about the situation meant that they did not feel ready to commit at the time of the very first approach, although that unease appears to have settled soon after arrival.”*  *24SC “However, other participants described feeling less welcoming of the initial approach, most often because they were feeling unwell, in pain or anxious. They seemed to describe feeling overwhelmed by what was happening”*  *24PC “Ms. H seemed to feel overwhelmed by being asked to read the patient information sheet very soon after her arrival, saying, “When I was asked to read I found it terribly difficult, in those circumstances, with your mind all weary with everything that happened so suddenly”.*  *36SC “it was the timing of the information which meant that the women were unable to consider it properly:”*  *36SC/PC “one woman who felt that there was too much information, but her response also reflects the inappropriate timing. In pain when she was asked to take part in the study she did not want to be given so much information because: “At that point, I couldn't read it. My husband read the information sheet] and I signed.”*  *36PC “I was first told about the study] in the labor suite. I feel the information should have been made available at pre-natal checks. It should be better if patients were asked to participate when not cup to high doh'. I strongly feel that I should, have had a chance to go home and discuss it with my husband. I wasn't really capable of making a decision and. my husband had to decide.”*  *36PC “I was offered an information sheet, but I was not feeling like reading it.*  *They gave me a leaflet, I think, but I just put it aside, I was in such pain.*  *I got more information when I was in labor, but I wasn't really in a fit state to pay attention I'd have said yes to anything, then.”*  *36SC “The protocol for the labor trial specified that one of the researchers would attend antenatal classes in order to inform patients and their midwives about the study, yet, as Table 3 shows, 88% of the women contended that they were not told about the trial until they were actually in labor. This is a very large percentage, and is supported by the women's comments:”*  *36PC “I first heard about it when I was taken down to the maternity room"*  *36PC “I didn't know anything about it until I was in labor ... It was not mentioned at antenatal classes.”*  *36PC “At an antenatal class, we were told about another study. There was no mention of [this] study.”*  *36PC “[The study was] first mentioned about 5 or 1A minutes before I was given the drugs]”*  *37SC “Women reported that they had been recruited to the ORACLE trial on arriving in hospital with premature labour or broken waters. They were potentially anxious, stressed and in pain. Despite staﬀ receiving careful training to talk to women and stress the importance of informed consent, and women being provided with an information sheet about the trial approved by the ethics committee, most of the women in our study described having made the decision to take part without much consideration.”*  *38PC “I had so many people around my bed, I don’t really remember. I had all sorts of people doing all sorts of things. It’s not like being asked to join a trial when you are well is it? You are being asked to join a trial when you could die, basically.*  *38PC “I recall him telling me what it was about but at the same time I had a paediatrician telling me what the odds of the baby surviving were.”*  *38PC “It may be good to be told about it at the beginning of the pregnancy rather than you giving birth and you are really poorly or you are on drugs so your head is not quite there.”*  *38PC “I really don’t think they should ask you when you are in the late stages of labour and about to give birth.”*  *43SC “In addition, most described recognising, with hindsight, that the approach to take part in the trial had taken place at a time when they had been in a very vulnerable emotional situation; one which had resulted in their consenting from ‘a point of desperation’ as Arlene aptly put. The women who felt this way described how, at the time they were recruited, they would have considered almost any option which might have prevented them from having to undergo further invasive medical procedures and from having to leave their baby:”* | |
| - - Excessive Volume(5) | *1SC “One participant felt overloaded with information from a number of different health professionals at a difficult and emotional time”*  *1SC “Trial information was given at a time when patients had a lot of information to take in about their diagnosis and treatment.”*  *1PC “It was almost at the time I was just starting chemo, so I had a load of information from the lung nurse, from the doctor… the specialist, uh, which quite honestly was almost an overload.”*  *1PC “Then I had this trial, which is another load of information, and it’s quite a bit of an overload when your mind is in [turmoil anyway].”*  *7SC/PC “In this cancer outpatient service, women were at different points in their identiﬁcation, adjustment and management of illness. Receiving new information about a diagnosis and/or options about cancer treatment left women to assimilate this information and their reactions to the details, while trying to participate effectively in care planning decisions. They felt bafﬂed with the amount of information and found it difﬁcult to concentrate during the consultation, picking up on only some bits of the information. ‘ … to tell you the truth I don’t know that day to this what the doctor, consultant I should call him, said to me because the only thing I picked up on was chemotherapy. “What you telling me?” and that was it. So … ’ (SN8, ovarian) Receiving an opportunity to take part in a trial at this point added to the complexity of this process, especially when the new treatments offered in the trial and standard treatments appeared similar, and impacted on how they reasoned about the different options. Women found it difﬁcult to differentiate between treatments offered as standard and within the trial.”*  *9SC “Many participants expressed that they were inundated with too much information and intimidated by details on side effects and the medical and legal language used in the consent process.”*  *36PC “[The doctor] gave us two A4 pages of written information but I was not in any state to read it. There was also] verbal information, but I was not up to understanding it.”*  *36SC/PC “one woman who felt that there was too much information, but her response also reflects the inappropriate timing. In pain when she was asked to take part in the study she did not want to be given so much information because: “At that point, I couldn't read it. My husband read the information sheet] and I signed.”*  *38PC “I had so many people around my bed, I don’t really remember. I had all sorts of people doing all sorts of things. It’s not like being asked to join a trial when you are well is it? You are being asked to join a trial when you could die, basically.”* | |
| - - Too Much Information Simultaneously(5) | *1SC “One participant felt overloaded with information from a number of different health professionals at a difficult and emotional time”*  *1PC “It was almost at the time I was just starting chemo, so I had a load of information from the lung nurse, from the doctor… the specialist, uh, which quite honestly was almost an overload.”*  *7SC/PC “In this cancer outpatient service, women were at different points in their identiﬁcation, adjustment and management of illness. Receiving new information about a diagnosis and/or options about cancer treatment left women to assimilate this information and their reactions to the details, while trying to participate effectively in care planning decisions. They felt bafﬂed with the amount of information and found it difﬁcult to concentrate during the consultation, picking up on only some bits of the information. ‘ … to tell you the truth I don’t know that day to this what the doctor, consultant I should call him, said to me because the only thing I picked up on was chemotherapy. “What you telling me?” and that was it. So … ’ (SN8, ovarian) Receiving an opportunity to take part in a trial at this point added to the complexity of this process, especially when the new treatments offered in the trial and standard treatments appeared similar, and impacted on how they reasoned about the different options. Women found it difﬁcult to differentiate between treatments offered as standard and within the trial.”*  *9SC “For example, participants often signed multiple consent forms including ones for transfusions and bone marrow biopsies, as well as informed consent forms for standard HSCT medical care and clinical research trials. This not only overwhelmed patients, but often confused them since the clinical research trial forms required that aspects of routine HSCT care also be discussed. Thus, patients saw the same information duplicated on multiple forms making unclear which aspects were routine and which were experimental”*  *38PC “I recall him telling me what it was about but at the same time I had a paediatrician telling me what the odds of the baby surviving were.”*  *38PC “I had so many people around my bed, I don’t really remember. I had all sorts of people doing all sorts of things.”*  *43SC “Some women, including Heather, also reflected on how their concentration and ability to take trial information on board had been further compromised by their awareness that they were in an emergency situation with a lot of activity taking place around them:”* | |
| - - Too Many Interlocutors(5) | *1SC “One participant felt overloaded with information from a number of different health professionals at a difficult and emotional time”*  *1PC “It was almost at the time I was just starting chemo, so I had a load of information from the lung nurse, from the doctor… the specialist, uh, which quite honestly was almost an overload.”*  *7SC/PC “In this cancer outpatient service, women were at different points in their identiﬁcation, adjustment and management of illness. Receiving new information about a diagnosis and/or options about cancer treatment left women to assimilate this information and their reactions to the details, while trying to participate effectively in care planning decisions. They felt bafﬂed with the amount of information and found it difﬁcult to concentrate during the consultation, picking up on only some bits of the information. ‘ … to tell you the truth I don’t know that day to this what the doctor, consultant I should call him, said to me because the only thing I picked up on was chemotherapy. “What you telling me?” and that was it. So … ’ (SN8, ovarian) Receiving an opportunity to take part in a trial at this point added to the complexity of this process, especially when the new treatments offered in the trial and standard treatments appeared similar, and impacted on how they reasoned about the different options. Women found it difﬁcult to differentiate between treatments offered as standard and within the trial.”*  *9SC “For example, participants often signed multiple consent forms including ones for transfusions and bone marrow biopsies, as well as informed consent forms for standard HSCT medical care and clinical research trials. This not only overwhelmed patients, but often confused them since the clinical research trial forms required that aspects of routine HSCT care also be discussed. Thus, patients saw the same information duplicated on multiple forms making unclear which aspects were routine and which were experimental”*  *38PC “I recall him telling me what it was about but at the same time I had a paediatrician telling me what the odds of the baby surviving were.”*  *38PC “I had so many people around my bed, I don’t really remember. I had all sorts of people doing all sorts of things.”*  *43SC “Some women, including Heather, also reflected on how their concentration and ability to take trial information on board had been further compromised by their awareness that they were in an emergency situation with a lot of activity taking place around them:”* | |
| - - Too Complex(3) | *7SC/PC “In this cancer outpatient service, women were at different points in their identiﬁcation, adjustment and management of illness. Receiving new information about a diagnosis and/or options about cancer treatment left women to assimilate this information and their reactions to the details, while trying to participate effectively in care planning decisions. They felt bafﬂed with the amount of information and found it difﬁcult to concentrate during the consultation, picking up on only some bits of the information. ‘ … to tell you the truth I don’t know that day to this what the doctor, consultant I should call him, said to me because the only thing I picked up on was chemotherapy. “What you telling me?” and that was it. So … ’ (SN8, ovarian) Receiving an opportunity to take part in a trial at this point added to the complexity of this process, especially when the new treatments offered in the trial and standard treatments appeared similar, and impacted on how they reasoned about the different options. Women found it difﬁcult to differentiate between treatments offered as standard and within the trial.”*  *30SC “However, many admitted that they could not remember or understand everything that was explained to them.”*  *38PC “I had so many people around my bed, I don’t really remember. I had all sorts of people doing all sorts of things. It’s not like being asked to join a trial when you are well is it? You are being asked to join a trial when you could die, basically.*  *38PC “I recall him telling me what it was about but at the same time I had a paediatrician telling me what the odds of the baby surviving were.”* | |
| - - Inadequate Format(5) | *1PC “P: Yeah, I got all the information sheets, but reading them [laughs]…. I: So you didn’t read them, you just went with what they said, basically. P: Yeah, if they are offering you something that they say might help, you have got to try everything you know.”*  *1SC “Many participants did not fully engage with the trial information prior to consenting to take part, no doubt explaining some of the limited understanding described above”*  *1SC “Most participants in the intervention group also chose not to engage in any depth with the written information”*  *1SC “This participant reported not to have read the trial information at all, relying only on the information given by the research nurse.”*  *24PC “Ms. H seemed to feel overwhelmed by being asked to read the patient information sheet very soon after her arrival, saying, “When I was asked to read I found it terribly difficult, in those circumstances, with your mind all weary with everything that happened so suddenly”.*  *36PC “[The doctor] gave us two A4 pages of written information but I was not in any state to read it. There was also] verbal information, but I was not up to understanding it.”*  *36PC “I was offered an information sheet, but I was not feeling like reading it.*  *They gave me a leaflet, I think, but I just put it aside, I was in such pain.*  *I got more information when I was in labor, but I wasn't really in a fit state to pay attention I'd have said yes to anything, then.”*  *38PC “I had so many people around my bed, I don’t really remember. I had all sorts of people doing all sorts of things. It’s not like being asked to join a trial when you are well is it? You are being asked to join a trial when you could die, basically. “*  *41PC “But maybe with hindsight I think it matters a lot: the person who comes to tell you [about the study]. If someone like Ms J [the research midwife] had come to my bed at the beginning and taken her time over it, then maybe I would’ve thought differently than when you have a doctor perched on the window-sill, only just not looking at her watch, saying I’m just popping in for five minutes and then I’m gone and you have to make a decision. Because you have to join the study at two o’clock this afternoon, otherwise it’s too late. Yes, that feels different and the results are different. Perhaps even when it comes to taking part in the research.”* | |
| - - Inconsiderate to Patient’s Capacity(5) | *24SC “Ms. C reported that a hearing impediment affected her ability to understand the explanations provided about the research.”*  *36PC “I was first told about the study] in the labor suite. I feel the information should have been made available at pre-natal checks. It should be better if patients were asked to participate when not cup to high doh'. I strongly feel that I should, have had a chance to go home and discuss it with my husband. I wasn't really capable of making a decision and. my husband had to decide.”*  *36PC “[The doctor] gave us two A4 pages of written information but I was not in any state to read it. There was also] verbal information, but I was not up to understanding it.”*  *36SC/PC “one woman who felt that there was too much information, but her response also reflects the inappropriate timing. In pain when she was asked to take part in the study she did not want to be given so much information because: “At that point, I couldn't read it. My husband read the information sheet] and I signed.”*  *36PC “I was offered an information sheet, but I was not feeling like reading it.*  *They gave me a leaflet, I think, but I just put it aside, I was in such pain.*  *I got more information when I was in labor, but I wasn't really in a fit state to pay attention I'd have said yes to anything, then.”*  *37SC “Women reported that they had been recruited to the ORACLE trial on arriving in hospital with premature labour or broken waters. They were potentially anxious, stressed and in pain. Despite staﬀ receiving careful training to talk to women and stress the importance of informed consent, and women being provided with an information sheet about the trial approved by the ethics committee, most of the women in our study described having made the decision to take part without much consideration.”*  *38PC “I had so many people around my bed, I don’t really remember. I had all sorts of people doing all sorts of things. It’s not like being asked to join a trial when you are well is it? You are being asked to join a trial when you could die, basically.*  *38PC “It may be good to be told about it at the beginning of the pregnancy rather than you giving birth and you are really poorly or you are on drugs so your head is not quite there.”*  *38PC “I really don’t think they should ask you when you are in the late stages of labour and about to give birth.”*  *43SC/PC “However, most also noted that, by the time they had been approached to take part, they had simply been too exhausted, distracted and/or emotionally overwhelmed to assimilate and retain all the information provided:*  *‘I genuinely think they probably told me everything about the trial, but my head was elsewhere, I was utterly exhausted.”*  *‘they (possible side effects of taking GTN) were on the form but if I’m being honest, I cannot remember what any of them were. I remember that the nurse read them out and went through it with me but I was utterly done in.’*  *43SC “In addition, most described recognising, with hindsight, that the approach to take part in the trial had taken place at a time when they had been in a very vulnerable emotional situation; one which had resulted in their consenting from ‘a point of desperation’ as Arlene aptly put. The women who felt this way described how, at the time they were recruited, they would have considered almost any option which might have prevented them from having to undergo further invasive medical procedures and from having to leave their baby:”* | |
| - - Essential information related to risk withheld or insufficient (5) | *2PC “I don’t know what other people need to know? It would be good for people to be told up front how bad the chemo is. How incredibly tired you feel. That can’t be said enough.”*  *7PC “It is easy to sort of understand what they were trying to achieve but as I said it doesn’t give you any information as to whether what they feel would be adequate for you, if it was on a banding system, whether it would actually be higher or lower than the toxins that you are getting now. That we don’t know”*  *7SC “Sometimes, it was felt that further details about the trial were only provided post consent. As one participant stated, she was told that the details about the trial will be explained if she decides to take part in the trial.”*  *7SC “Most participants regarded the consent information as easy to understand and the trial well explained, yet lacking in detail about the trial treatment options and their consequences. Information about the options and their consequences was seen as necessary to make an informed decision. For instance, details about the dose of the drug, the nature and likelihood of experiencing side effects and personal relevance of the trial tended not to be described.”*  *23SC “A negative aspect of the trial was lack of sufﬁcient explanation and discussions with the doctors”*  *36SC “Those patients who indicated that they had not received enough information were asked to describe the kind of information they would like to have received. Suggestions included more information about the way the drug worked, about possible side-effects, alternative treatment options, and potential side-effects”*  *38SC/PC “Women recruited to the Magpie Trial had noticeably differing requirements regarding the amount and content of trial information they sought. However, generally they felt that they did not receive adequate explanation and a sufﬁcient level of detail about the trial prior to joining:*  *“I didn’t know what it was for, whether it was to bring my blood pressure down. No one even mentioned ﬁtting that was completely new to me. It is the details of actually what it is for more than what it does.”*  *“I think they should have gone through it a little bit more to explain what would happen and a bit more information about the trial - no one really said it would stop convulsions or it would stop you from developing high blood pressure, they just did not explain it very well.”*  *Women discussed several issues regarding information provision and gave suggestions of what they would have liked to receive. These included the following: what the aim of the trial was, what it was hoping to ﬁnd out in relation to pre-eclampsia, how the trial treatment might work, the possible risks of joining, and how the trial would be administered.”* | |
| - Patient’s Follow Up(11) | Factors related to the follow up and the various activities such as visits, questionnaires and procedures required of patients during trial follow up | |
| - - Trial Visits badly organized and inconveniently timed(6) |  | |
| - - - Bad organization(3) | *1PC “The things that have annoyed me so far is you go to the [name of principal hospital in city 3], you’ve got to be there for eight o’clock in the morning, they got no bed for you. So they can’t do anything until they have found a bed for you, and I’ve been there from eight until half past one, um, just hanging around.”*  *1SC “Adding to this, the patient also lived a considerable distance from the hospital, and it seems he incurred considerable expense and inconvenience every time he attended an appointment, some of which he also felt to have been unnecessary or badly managed.”*  *55PC “Okay, not very good. Lengthy procedures and long term follow up. Too frequent visits and time consuming”*  *C PC/SC “they were dissatisfied with the extended waiting times in clinic:*  *I go upstairs straight away and take my card. Ahhh I go, then get called to give some bloods, and then um, I go back into the waiting room and it’s about on a good day it’s about uh 20 minutes to half, but we get there, I have my blood taken, see the consultant, then I have to wile away two hours. The only complaint I’ve got is getting my treatment. There’s, the prescription is never ready so I always have to wait for the nurse to go to get the prescription because either the doctor doesn’t send it down or pharmacy are haven’t done it and they have to wait to get it checked. Quite often we’re there for a good two hours whereas my treatment only takes half an hour. (U03, urban area, IV treatment)”* | |
| - - - Irrelevant or Unnecessary visits(2) | *1SC “Adding to this, the patient also lived a considerable distance from the hospital, and it seems he incurred considerable expense and inconvenience every time he attended an appointment, some of which he also felt to have been unnecessary or badly managed.” (CUT)*  *1SC “This patient also understood there to be greater costs to trial participation. The patient had recently learned that he could be treated with surgery instead of chemotherapy, which meant that he would need to make additional non-treatment-related visits to the hospital to fulfill his role as a trial participant.”*  *55PC “Okay, not very good. Lengthy procedures and long term follow up. Too frequent visits and time consuming”* | |
| - - - Inconvenient timing of visits(4) | *1PC “The things that have annoyed me so far is you go to the [name of principal hospital in city 3], you’ve got to be there for eight o’clock in the morning, they got no bed for you. So they can’t do anything until they have found a bed for you, and I’ve been there from eight until half past one, um, just hanging around.”*  *1SC “Adding to this, the patient also lived a considerable distance from the hospital, and it seems he incurred considerable expense and inconvenience every time he attended an appointment, some of which he also felt to have been unnecessary or badly managed.”*  *4SC “participants identified travel distance, timing of the SMAs, and being too sick to travel as reasons for lack of attendance.”*  *6SC “patients had to attend early morning appointments. Therefore, they had to travel to appointments in rush hour traffic”*  *A SC “Some participants had the feeling that they had to adapt their agenda to those of the professionals instead of the other way around.”*  *A PC ‘I had to train three times a week. I was surprised by the fact that professionals presume that you have plenty of time. The training was only possible on some days, but they did not take my possibilities and preferences into account.’* | |
| - - - Great travel distance with parking difficulties(4) | *1PC “Like I say, difficult parking and all the rest of it, I took part in so much as went along with all that was asked me”*  *1SC “Adding to this, the patient also lived a considerable distance from the hospital, and it seems he incurred considerable expense and inconvenience every time he attended an appointment, some of which he also felt to have been unnecessary or badly managed.”*  *4SC “participants identified travel distance, timing of the SMAs, and being too sick to travel as reasons for lack of attendance.”*  *6SC “A number described the frustrations of being unable to find a car parking space on their arrival.”*  *55PC “Very bad. Very time consuming, far away distance.”* | |
| - - Trial Questionnaires too frequent, lengthy or challenging(7) |  | |
| - - - Demanding Frequency(3) | *3BPC “I thought oh crumbs [laughter]. I thought oh God every day.”*  *13SC “The challenge of ‘completing the questionnaires’ was explored as participants had to complete a number of questionnaires throughout the trial”*  *51PC “I am not too keen on questionnaires but I know how important they are to research.”*  *“Having to make a day-to-day diary and filling in forms and such like.”*  *“All the writing (which is done by my wife!).”*  *“I would have favored a weekly diary rather than a daily one.”* | |
| - - - Tedious Duration(2) | *34SC “A second participant felt that the questionnaire was too long and time consuming and this discouraged her from responding.”*  *35SC “However, a minority of patients did say they struggled with the length of some of the questionnaires.*  *35PC “It [questionnaire] was hard work because there were so many pages.”* | |
| - - - Assignments too Challenging(5) | *3BSC “Many participants expressed initially feeling daunted upon receiving the packs of outcome measures to be completed during the trial.”*  *13SC “The challenge of ‘completing the questionnaires’ was explored*  *as participants had to complete a number of questionnaires throughout the trial”*  *13SC “Some reported their fatigue levels ﬂuctuated making the questionnaires challenging to complete.”*  *34PC “Some of the ‘how do you feel at this present time’ with your state of mind . . . Things I didn’t think they were relevant to a foot injury erm so some of them were a bit mmm I don’t know a bit difﬁcult.”*  *A SC/PC “The communication about the questionnaires that needed to be filled in was sometimes not sufficient. Some participants missed some explanation about the content and character of the questionnaires. The lack of information sometimes led to frustration and anger:*  *‘Don’t repeat the question that often. It made me feel irritated.’ (FSHD)*  *‘The questions irritated me and my son, enormously! ‘I would commit suicide if I had the possibility’ is outrageous! My son has thrown away everything and he will never participate in a study from this hospital again. (….) Whose ideas are these questions? Are they from a researcher who doesn’t have a clue about the possible effects of these questions?!’ (FSHD)”*  *C SC/PC “Many of the interviewees were dissatisﬁed either with the questionnaires and scales that they had to complete or the strategies they used to complete them. They complained about the difﬁculties of expressing complex experiences in the standardised terms the questionnaire asked of them.*  *Questionnaires are always terrible because you never can express by checking a box what one wants to say. [QG2/ 241]*  *If I make this movement, it hurts here. If I make that movement, it hurts there. Now the pain is gone. Now I look at you and I don’t experience any pain. Now you tell me, do I have pain or do I not have pain? You tell me! [QG2/318]”*  *C PC/SC “Furthermore, the questionnaires used in the study left some women feeling uneasy and unhappy with their contribution.*  *‘I was actually glad when I was done, just like school work that I had to do and I did very thoroughly. But I was not satisﬁed with my work and also not with the questions! So, I wasn’t– but I have experienced such feelings with other questionnaires before. [QG2/265] ‘*  *‘I hope I have ﬁlled out everything correctly. I do not know if I ﬁlled them out correctly. [PN9/163]’*  *One woman called a family member and her family physician to assist her in completing the questionnaire in order to ensure the correctness of the questionnaires.*  *‘I don’t remember for which question that was. I really did not know what to do with that question. I did not want to do anything wrong, so I called my daughter. She is a teacher and she also really had to think about it. But I cannot tell you which question that was at the time. I don’t know. But the question was phrased very strange.’ [QG8/207]”* | |
| - - Physical capacity to attend visits or complete questionnaires not considered (2) | *13SC “Some reported their fatigue levels ﬂuctuated making the questionnaires challenging to complete.”*  *4SC “participants identified travel distance, timing of the SMAs, and being too sick to travel as reasons for lack of attendance.”* | |
| - Trial Closure(5) | Factors related to the end of the trial and closure process | |
| - - Lack of feedback related to treatment allocation and results(5) | *2PC “It would be good to know where they went with this stuff. What did they ﬁnd? Did they keep going? Years from now, even if I’m gone, I’d love for my family to get a newsletter from _____ telling me the results of the trial.”*  *9SC “A common concern among several participants, both adult patients and parents of pediatric patients, pertained to the uncertainty about the outcome of the study and the lack of feedback patients received once they enrolled and completed the study.”*  *13SC “Control group participants wanted to know study ﬁndings:”*  *13PC “…liked to have known if acupuncture helped the group who had the treatment… did it work or not? (P2NCG). I would like to talk to people who had it[acupuncture] done (P1NCG).”*  *44SC “Most women (24 out of 38) explicitly indicated during interviews that they would like to know their individual treatment allocation. This desire was motivated by a range of reasons. For some participants (6), it was framed in terms of general curiosity.”*  *44PC “It would be intriguing to know which bit of the study I’d been in, whether I’d actually had the drugs or whether I’d had placebo or whatever, it’d be quite interesting to know.”*  *44PC “I think it’s just curiosity really. I wanted to know whether I was on the antibiotic or the placebo one, that’s all I wanted to know.”*  *A SC/PC “The lack of communication about the individual trial results was, at last, sometimes disappointing for participants:*  *‘It’s a pity that I haven’t heard anything about the final measurements but I was just another patient for him [the psychologist]. I think it’s more important for me than it is for him. He had promised me to give me the results, but I haven’t heard anything.’(PPS)*  *‘It would have been nice if I had heard my own progression. Why isn’t that possible? ‘ (FSHD)”* | |
| **SUBTHEME: Burdensome Impacts and Consequences(28)** | **The negative psychological, physical and cost impacts experienced by patients through trial participation** | |
| - Cost Impacts(1) | The economic impacts and costs of trial participation | |
| - - Travel expenses incurred due to considerable travel distances(1) | *1SC “the patient also lived a considerable distance from the hospital, and it seems he incurred considerable expense and inconvenience every time he attended an appointment”* | |
| - Physical Impacts(11) | The negative physical impacts and consequences experienced by patients due to their trial participation | |
| - - Time consumed by lengthy duration of trial and related tasks(visits, activities, procedures)(8) | *1PC “The things that have annoyed me so far is you go to the [name of principal hospital in city 3], you’ve got to be there for eight o’clock in the morning, they got no bed for you. So they can’t do anything until they have found a bed for you, and I’ve been there from eight until half past one, um, just hanging around.”*  *2SC “Personal belief they have little time left and their time would be shortened by participating in a clinical trial.”*  *6SC “The long duration of the clinic appointments (sometimes lasting the whole morning”*  *24SC/PC “Ms. A believed that participation in the research caused her to stay longer in hospital, saying, “I think because they might have done an extra test I think I had to wait longer”.*  *34SC “A second participant felt that the questionnaire was too long and time consuming and this discouraged her from responding.”*  *51SC “However, a small number of participants stated a dislike about the duration and commitment required of them during their participation of the trial (n=6). It was suggested that a study for a six-month period would have been preferable and more convenient to them.”*  *51PC “The length of time the survey carried on for and the commitment necessary to see it through.”*  *51PC “Perhaps the length of your study. A six-month study might have been better. Most volunteers went on holiday during the year and you feel having to take tablets while you were away was an inconvenience. In my case I was in Australia.”*  *55PC “Okay, not very good. Lengthy procedures and long term follow up. Too frequent visits and time consuming”*  *55PC “Very bad. Very time consuming, far away distance.”*  *CSC/PC” they were dissatisfied with the extended waiting times in clinic: I go upstairs straight away and take my card. Ahhh I go, then get called to give some bloods, and then um, I go back into the waiting room and it’s about on a good day it’s about uh 20 minutes to half, but we get there, I have my blood taken, see the consultant, then I have to wile away two hours. The only complaint I’ve got is getting my treatment. There’s, the prescription is never ready so I always have to wait for the nurse to go to get the prescription because either the doctor doesn’t send it down or pharmacy are haven’t done it and they have to wait to get it checked. Quite often we’re there for a good two hours whereas my treatment only takes half an hour. (U03, urban area, IV treatment)”* | |
| - - Time wasted by taking a placebo(2) | *1SC “Although he understood the principle of randomisation, he felt that his expectations had been raised and his time wasted.”*  *1PC “I mean, well, as far as I was concerned, it was a waste of time.”*  *51PC “Having wasted a year taking the placebo, I would have preferred if it had been 6 months vitamins and minerals and 6 months placebo, but still a blind test.”* | |
| - - Worsening of symptom/disease burden completing trial questionnaires(5) | *3BPC “Some of the days when I was really, really poorly with the chemo I couldn't even get out of bed to even fill it in I’ll be honest, but I knew each day how bad I was, how sick I was. So, you know, was able to fill it in accurately because I knew that at the beginning I was so poorly and so sick and by the end of it you are…”*  *3BSC “In contrast a small number of participants indicated they had experienced some negative effects from completing the outcome measures, chiefly that reading and rating their level of nausea and vomiting at a time when they were feeling nauseous had at times worsened their experience.”*  *4SC “participants identified travel distance, timing of the SMAs, and being too sick to travel as reasons for lack of attendance.”*  *13SC “Some reported their fatigue levels ﬂuctuated making the questionnaires challenging to complete.”*  *13PC … got a bit confused (P2S) and I just kept misreading them … (P2NO).*  *51PC “Being already on a lot of pills, my dislike was having to take more.”* | |
| - Psychological Impacts(27) | The negative cognitive and emotional consequences experienced by patients due to trial participation | |
| - - Informed Consent Process(14) |  | |
| - - - Anxious to participate in a trial(1) | *24SC “Some interviewees described being generally skeptical of clinical research and initially felt anxious about participation.”* | |
| - - - Afraid to be used as a ‘guinea pig’(4) | *2SC “For example, approximately half of the patients interviewed said they feared clinical trials for the following reasons:*  *-Being a guinea pig—being used to further the career of a scientist without beneﬁt to one’s present condition,*  *-Assuming all clinical trials involve a placebo and patients “runs the risk” of receiving the placebo (i.e., getting no treatment) and dying sooner,*  *-Personal belief they have little time left and their time would be shortened by participating in a clinical trial.”*  *9SC/PC “Additionally, both adult patients and parents mentioned the uncertainty associated with clinical trial outcomes and general concerns with participation in trials, providing comments such as “guinea pig,” “lab rat,” and “we are one step above the mice.”*  *46SC/PC “In contrast, trials testing new drugs were perceived as risky, making the impression of “acting as a guinea pig” – with negative effect on willingness to participate in these trials.”*  *49SC “The patients’ attitude ranged from research being very important to a sceptical view that doctors used patients as guinea pigs for the doctors’ own benefit.”* | |
| - - - Afraid to not be treated(1) | *30PC “‘It depends on the doctors. If I said I was afraid to participate, they might not treat me’. ‘I would get treatment from hospital. Everyone is getting treatment here’.* | |
| - - - Intimidated and confused by scientific and medico-legal terminologies(5) | *1PC “You don’t need to know the terminology and all that, because when you look it up on the Internet, it’s frightening.”*  *9SC “Additionally, many participants repeated their concerns about the amount of paperwork and the scientific and legal language involved in the consent process.”*  *9SC “Many participants expressed that they were inundated with too much information and intimidated by details on side effects and the medical and legal language used in the consent process.”*  *21PC “He also felt that some of the information was “too technical. I am probably above average intelligence...Others would surely struggle with the information.”*    *38SC “For women who had difﬁculty comprehending the trial information, the language the midwives or obstetricians used to explain the trial appeared to be the cause of misunderstandings. Women stated that clinicians provided oral information that was too technical for them to grasp; and that the concepts used by the clinicians were often unfamiliar. Women wanted the information to be in clear, simple, lay language and presentation:*  *38PC “I suppose if he (the doctor) had simpliﬁed it. The doctor who was telling us gave us this sales pitch in the words he had obviously come to use and when he hit a blank wall he did not know how to explain it in a more plain English way. What I had to do to make my decision was I had to ask him: ‘the way you have explained this trial to me, I think it is x, y z am I right?’ and I actually had to ask him ‘is this what you are telling me?’ I actually had to come up with my own way of interpreting what he said.”*  *38PC “Not that I could really understand the doctor. It was the midwife really who explained it to me she explained it sort of in our words, you know, because doctors give you all these big words don’t they that you don’t understand?”*  *53PC “I understood bits of it, some things I didn't understand. The second time I went I took my daughter with me. She explained what he said and that they will offer to get somebody to translate for me. When I visit the doctor I occasionally take my daughter because of the terminology used.”*  *53SC “Difficulties with understanding clinical trial terminologies. Difficulties with understanding clinical trial terminologies was an issue that came up frequently, in particular for the older South Asian respondents, where lack of fluency in the English language led to uncertainty and confusion.”* | |
| - - - Cognitively overwhelmed and stressed by trial information overload(8) | *1PC “Then I had this trial, which is another load of information, and it’s quite a bit of an overload when your mind is in [turmoil anyway].”*  *1SC “This patient’s response to ‘information overload’ was thus to ‘switch off’ or disengage.”*  *1SC “Information about the trial added to both his information and stress burden.”*  *2PC “I’m the type of patient you can give too much information to—you can put me off the process if it requires me to listen too much or make too many decisions.”*  *7PC “… to tell you the truth I don’t know that day to this what the doctor, consultant I should call him, said to me because the only thing I picked up on was chemotherapy. “What you telling me?” and that was it. So …”*  *7SC “They felt bafﬂed with the amount of information and found it difﬁcult to concentrate during the consultation, picking up on only some bits of the information.”*  *9SC “Many participants expressed that they were inundated with too much information and intimidated by details on side effects and the medical and legal language used in the consent process.”*  *24PC “Ms. H seemed to feel overwhelmed by being asked to read the patient information sheet very soon after her arrival, saying, “When I was asked to read I found it terribly difficult, in those circumstances, with your mind all weary with everything that happened so suddenly”.*  *30SC “However, many admitted that they could not remember or understand everything that was explained to them because of their malaria symptoms during the informed consent process.”*  *36PC “[The doctor] gave us two A4 pages of written information but I was not in any state to read it. There was also] verbal information, but I was not up to understanding it.”*  *36SC/PC “one woman who felt that there was too much information, but her response also reflects the inappropriate timing. In pain when she was asked to take part in the study she did not want to be given so much information because: “At that point, I couldn't read it. My husband read the information sheet] and I signed.”*  *41SC “In both groups women indicated that being confronted with an (unexpected) invitation to participate in scientific research was stressful and needed thorough consideration.”* | |
| - - - Embarrassed to admit not understanding trial information(1) | *21SC “the possibility that some other patients may have been too embarrassed to admit to the investigator that they had not been able fully to understand the information.”* | |
| - - - Pressure to participate(3) | *9SC “Finally, some adult patients felt somewhat pressured by their physicians to participate in certain clinical research studies.*  *12PC “Mr. Chase: Didn’t know which other way to go. I found it an immensely difﬁcult decision to make.”*  *12SC “The men struggled with competing views but eventually had to decide whether or not to participate in the trial.”*  *12PC “Mr. Smith: To feel that this very important decision, which is genuinely a decision about the possibility of life or death at some point in the future, being down to chance”*  *53SC “One respondent signed the consent form as a result of indirect social pressure watching others sign.”*  *53PC “I’m not sure. There was another Asian women who signed the form so I copied her. I thought if she’s signed it, it must be okay. The other lady understood English I didn’t know that much.”* | |
| - - Randomized allocation process(9) |  | |
| - - - Discomfort and discontent to be randomized(2) | *10SC “A general discomfort with the randomisation in itself was articulated across interviews.*  *10SC “They strongly expressed unease and discontent with not just ‘‘getting it’’ and having to accept a drawing of lots (ie, randomisation),”*  *10PC “… deep inside I believed the new treatment being better, and now I accepted to participate in a drawing of lots. I was not certain of winning the draw, if you understand? If I had got the standard treatment I maybe would have felt… it’s not as good as the one I’ve got. I think its wrong …”*  *10PC “…They spoke highly about that chemo I got. However, they can just give it to me. Why must I be put in a pool, [of patients] and then toss a coin about it? … I just think that the drawing of lots is strange. Why can’t they just give you the one they think is the most effective? I did not understand … [patient 2 (breast cancer, trial participant, CEF)]”*  *34SC/PC “For example, the following quote comes from a participant who was unhappy with the treatment she was randomized to. “And it’s not been a priority and partly as well because yes I didn’t I was annoyed that I felt that I was not . . . getting didn’t have the treatment . . . that I would have liked . . . that kind of made me a bit reticent to make time””* | |
| - - - Disappointed, angered and depressed by control arm allocation(4) | *1SC “there may be negative psychological and emotional consequences of allocation to the control arm in non-placebo trials, such as disappointment and hopelessness.”*  *1SC “The exception, by contrast, was so disappointed and angered by the outcome that he withdrew from the trial shortly after we interviewed him and did not participate.”*  *3BSC “However, despite this approach many allocated to treatment as normal still indicated they felt ‘disappointed’ at not receiving wristbands.”*  *4SC “A control patient expressed disappointment about not being randomized to the intervention group and therefore did not get the added support that may have been available through the trial.”*  *13SC “For those patients who were part of the control group, there was disappointment on ‘hearing the news’”*  *13PC “Another participant when told she was in the control group reported feeling there was: …no hope for me… extremely depressed …went home and cried. To leave the hospital after an hour of filling forms … empty handed. I felt no one really understood how bad I felt”*  *13PC “I was disappointed … just selection isn't it? (P2NCG).* | |
| - - - Anxious and afraid to receive placebo(2) | *2SC “For example, approximately half of the patients interviewed said they feared clinical trials for the following reasons:*  *-Being a guinea pig—being used to further the career of a scientist without beneﬁt to one’s present condition,*  *-Assuming all clinical trials involve a placebo and patients “runs the risk” of receiving the placebo (i.e., getting no treatment) and dying sooner,*  *-Personal belief they have little time left and their time would be shortened by participating in a clinical trial.”*  *51PC/SC “Some MAVIS participants reported having issues with the possibility of receiving a placebo, as they didn’t know what treatment they had been allocated to” “I would have excluded the placebo.”*  *“Not knowing if I was taking a vitamin or a dummy.”*  *“Wondering if it was placebo.”*  *“No - although a bit wary not knowing whether the tablet was a 'smarty' or the real thing.”* | |
| - - - Embarrassed to benefit from placebo(2) | *3BPC “but if you went for a meal or if you were out somewhere socially where people didn’t know you, you would feel slightly embarrassed.”*  *3BPC However, a few revealed that they had inhibitions about wearing them in the company of others; or had received negative responses from others to their wearing of the wristbands.*  *3BPC “It’s a silly point but they are there all the time, so you do sort of start pulling your sleeves down because you don’t want people to see them, you know it looks rather strange walking round with two wristbands*  *18SC “For example, Abigail, a retired teacher in her 60s, talked about how she would feel pretty stupid, as if she made it up, if she found she had benefited from placebo acupuncture.”* | |
| - - Patient’s Follow Up(10) |  | |
| - - - Annoyed by bad organization of trial visits(1) | *1PC “The things that have annoyed me so far is you go to the [name of principal hospital in city 3], you’ve got to be there for eight o’clock in the morning, they got no bed for you. So they can’t do anything until they have found a bed for you, and I’ve been there from eight until half past one, um, just hanging around.”* | |
| - - - Endure intrusive interactions(1) | *2PC “I don’t like talking about my cancer with other people. I don’t like answering their questions or sharing that information about myself.”*  *2SC “The other half felt their medical information was private and not appropriate to talk about with others outside the family.* | |
| - - - “Feel like a patient every day” (1) | *4SC/PC “Similarly participants who were feeling relatively well or preferred to use denial or avoidance to cope with their illness didn't want to feel like “...a patient every day”*  *4PC “I did sort of let go for a while on participation, and it was more because I was having too much fun, and I didn't want to be a patient that day. I don't want to be a patient every day of my life. And, so the less time I have with the medical profession, the more I feel like a normal person.”* | |
| - - - Questionnaire frustration and dissatisfaction(2) | *A PC/SC “The communication about the questionnaires that needed to be filled in was sometimes not sufficient. Some participants missed some explanation about the content and character of the questionnaires. The lack of information sometimes led to frustration and anger:*  *‘Don’t repeat the question that often. It made me feel irritated.’ (FSHD)*  *‘The questions irritated me and my son, enormously! ‘I would commit suicide if I had the possibility’ is outrageous! My son has thrown away everything and he will never participate in a study from this hospital again. (….) Whose ideas are these questions? Are they from a researcher who doesn’t have a clue about the possible effects of these questions?!’ (FSHD)”*  *C PC/SC “Many of the interviewees were dissatisﬁed either with the questionnaires and scales that they had to complete or the strategies they used to complete them. They complained about the difﬁculties of expressing complex experiences in the standardised terms the questionnaire asked of them.*  *Questionnaires are always terrible because you never can express by checking a box what one wants to say. [QG2/ 241]*  *If I make this movement, it hurts here. If I make that movement, it hurts there. Now the pain is gone. Now Ilook at you and I don’t experience any pain. Now you tell me, do I have pain or do I not have pain? You tell me! [QG2/318]”*  *C PC/SC “Furthermore, the questionnaires used in the study left some women feeling uneasy and unhappy with their contribution.*  *‘I was actually glad when I was done, just like school work that I had to do and I did very thoroughly. But I was not satisﬁed with my work and also not with the questions! So, I wasn’t– but I have experienced such feelings with other questionnaires before. [QG2/265] ‘*  *‘I hope I have ﬁlled out everything correctly. I do not know if I ﬁlled them out correctly. [PN9/163]’*  *One woman called a family member and her family*  *physician to assist her in completing the questionnaire in order to ensure the correctness of the questionnaires.*  *‘I don’t remember for which question that was. I really did not know what to do with that question. I did not want to do anything wrong, so I called my daughter. She is a teacher and she also really had to think about it. But I cannot tell you which question that was at the time. I don’t know. But the question was phrased very strange.’ [QG8/207]”* | |
| - - - Sense of obligation to the trial(5) | *23SC “One participant responded that feeling a responsibility to ﬁnish the trial was another motivation that made them follow the strict protocol.”*  *29SC “Even though participants conveyed this sense of responsibility towards the integrity of the trial,”*  *30SC “In addition, most subjects (N = 15) were not aware of their right to quit the research anytime if they wished to do so. They rather wanted to continue after being enrolled as they had committed to this at the beginning. They also felt obliged to adhere to the study until the end so that they would get complete recovery from their malaria.”*  *32SC “The participants internalized their obligation to engage with the trial but some simultaneously felt as if they never did enough”*  *32SC “When the participants completed the trial and reﬂected on their level of engagement during the interviews, most of them said that if they were to participate in the trial all over again that they would ‘‘try harder.’’ They felt guilty for their lack of commitment to their obligations to the trial.”*  *34SC/PC Another prevalent motivator for participants to respond was the fact that they had agreed to take part in the trial and felt that they should see it through. This is reﬂected in comments such as: “I said I would (ﬁll in questionnaire) so I stuck to my word.”* | |
| - - Trial Closure(2) |  | |
| - - - Sense of loss and isolation, disappointment and anxiety due to trial closure(2) | *6SC “Nonetheless, a sense of loss and isolation was still apparent.”*  *6SC “Several patients described feeling alone with their diabetes, an experience brought about by having less structured and frequent contact with medical professionals”*  *6PC “Unsurprisingly, patients were disappointed when the UKPDS ended.”*  *­6PC ‘It was a bit of a blow’; ‘In a way I suppose I was a bit anxious’; ‘I felt as though I was losing friends here’.*  *APC/SC “Participants at last mentioned a lack of control regarding the transition to regular care after the RCT intervention:*  *‘The transition can be better. I think the intervention stops too promptly. Of course you are aware that there will be an end and you should do something yourself, but I think that the organisation should also arrange a transition to regular care. They must do everything reasonably possible to provide care afterwards.’”* | |
| **THEME: Trial participation benefits (23)** | **Positive beneficial impacts experienced by patients through trial participation** | |
| Altruistic Benefits (10) | Beneficial effects of participation as a rewarding pro-social behavior | |
| - It ‘feels good’ to do good (5) | *1SC “In later interviews, several patients reflected on some of the benefits that they had experienced from taking part. As described above, many patients expressed altruistic motives for participation, and one of these described what could be considered a ‘feel-good’ factor which he gained from participation, particularly given the minimal commitment needed.”*  *1SC “reasons for taking part were to contribute to cancer research that might help other patients in the future. These participants seemed to appreciate the value of research for advancing treatments, and there was a sense that participating in research was the right thing to do.”*  *3BSC “A number felt that they had experienced positive outcomes as a result of taking part in the trial and this linked to their reports of motivations for taking part. Most frequent was a sense of wellbeing as a result of feeling they had completed an altruistic act and helped others.”*  *3BPC “I think taking part in the trial is quite, it makes you feel better actually because it is a useful tool and it’s going to be of use for other people in the future…Yes because it makes you feel better doesn’t it if you feel you are contributing something.”*  *24SC/PC “remarked that participation “makes you feel useful” and said, “It gives satisfaction to take part in a trial”.*  *37SC “This enabled women to continue to feel good about having taken part; they experienced the warm glow of having helped others.”*  *37PC “Just the thought that something that you’ve done might help somebody in your position in the futures a really good feeling.”*  *40PC “Felt good to be a part of something bigger’* | |
| - Opportunity to contribute to future research and help future patients (11) | *1PC “What I’ve got is going to kill me anyway. There’s no cure. It might give me time but that’s about all….If something can be done to help people in the future, then surely that’s got to be a worthwhile thing when it’s not taking an awful lot of time and it doesn’t take, well, it doesn’t take anything.”*  *3BPC “I think taking part in the trial is quite, it makes you feel better actually because it is a useful tool and it’s going to be of use for other people in the future…Yes because it makes you feel better doesn’t it if you feel you are contributing something.”*  *3BSC “There was an appreciation that the willingness of previous patients to engage in research had improved their own cancer treatment, and that for cancer treatments to improve in the future current patients would need to take part in research studies. In particular, it was the altruistic act of attempting to improve the care of future cancer patients which motivated participants to assist in evaluating if acupressure wristbands were effective for chemotherapy related nausea.”*  *3BPC “My reasons were as I’d had various treatments obviously associated with the diagnosis I’d had earlier that year it was partly that each time I had treatment I did reflect on people that had gone before me who from their experience and their participation in possible research that that perhaps made things easier for me in my experience. So it was a small way of trying to help for the future. That was kind of in my head, that I just wanted to try and be part of this and hopefully that would help people like me in the future.”*  *4PC “Two usual care participants also commented about altruistic motivations: “Well, if what I've gone through can help anyone else, I don't mind” and “I just hope that my responses might help those that come along, you know, the newly diagnosed ones.”*  *4PC “This altruistic benefit is exemplified in the following: “I feel if I can help one person that gets cancer; the whole journey has been worth it.”*  *4SC “Several participants described wanting to help other patients by increasing health care providers and scientists understanding about the patients’ illness experience.”*  *24PC ““If people don’t take part in these things then nothing moves forward”*  *35PC “Well I just thought that anything that might help people in the future. You know if people don’t do things like that [then] improvements might take place but probably a lot more slowly. I think that it’s a good thing to do for the beneﬁt of people who are having operations in the future really that’s why.”*  *“I feel if I can help other people from my experiences then all well and good. That’s what it’s about so as I say how are they gonna learn if you can’t learn from other people.“*  *“My attitude to that [trial involvement] is things they know now somebody has done that in the past so if they learn more it’s going to be valuable to people in the future. I’m all for that... Years ago they used to cut peoples legs off with saws, they’ve moved on quite a bit from that haven’t they.“*  *“If I can help somebody else because somebody had probably done something before me that could help me.”*  *35SC “A desire to help future patients was seen as part of a wider system of reciprocal ‘social exchange’ that included reﬂection on the past as well as the future: patients expressed gratitude to unknown members of previous generations who had participated in research that had contributed to improvements in medical care that they were currently receiving.”*  *37PC “I think it’s important to do these things ‘cos it’s like any trial, if people aren’t willing to participate then it’s very diﬃcult to move forward. As hard as that may seem if some people have had a negative outcome, it’s better to push things forward than always be afraid almost to do anything”*  *41PC “And I also looked at it like this: these are studies for the future, and after all I have a daughter and you never know. In that case I’m the kind of person to take part in things for other people, so that it’s better in the future than it is now, for example. What other people have done in the past, I’m making use of now.”*  *49PC “If it can make it easier for somebody in the future, count me in.”*  *51PC “Yes, if it was going to help others.”*  *“Yes, because I like to think such research studies make meaningful contributions to medical knowledge and science.”*  *“Yes, if the research will help elderly people in the future, then the study is worthwhile.”*  *A PC/SC “Others mentioned altruistic reasons. By participating they had the hope to get a solution for the next generation(s):*  *‘I’m not going to recover or improve. I have to learn to live with it, but I can help other people. Maybe there will be a treatment for them in the future.’”*  *A PC/SC “Participants with an inherited disease mostly participated, because they wanted to do something for their own children who have or might have the same disease:*  *‘We have 3 children and they have 50% chance of having the same disease. It will take years before this study may give results. That will be too late for me but not for my children!’”* | |
| - A chance to ‘pay it forward’ and reciprocate the contribution made by previous generations of research participants (4) | *3BSC “There was an appreciation that the willingness of previous patients to engage in research had improved their own cancer treatment, and that for cancer treatments to improve in the future current patients would need to take part in research studies. In particular, it was the altruistic act of attempting to improve the care of future cancer patients which motivated participants to assist in evaluating if acupressure wristbands were effective for chemotherapy related nausea.”*  *3BPC “My reasons were as I’d had various treatments obviously associated with the diagnosis I’d had earlier that year it was partly that each time I had treatment I did reflect on people that had gone before me who from their experience and their participation in possible research that that perhaps made things easier for me in my experience. So it was a small way of trying to help for the future. That was kind of in my head, that I just wanted to try and be part of this and hopefully that would help people like me in the future.”*  *35SC “A desire to help future patients was seen as part of a wider system of reciprocal ‘social exchange’ that included reﬂection on the past as well as the future: patients expressed gratitude to unknown members of previous generations who had participated in research that had contributed to improvements in medical care that they were currently receiving.”*  *37PC “I think it’s important to do these things ‘cos it’s like any trial, if people aren’t willing to participate then it’s very diﬃcult to move forward. As hard as that may seem if some people have had a negative outcome, it’s better to push things forward than always be afraid almost to do anything”*  *41PC “And I also looked at it like this: these are studies for the future, and after all I have a daughter and you never know. In that case I’m the kind of person to take part in things for other people, so that it’s better in the future than it is now, for example. What other people have done in the past, I’m making use of now.”* | |
| - A way to give something back to the health care service and society (3) | *3BSC “Undoubtedly the main motivational factor influencing participants was a desire to ‘give something back’.”*  *4PC “Most intervention and usual care participants expressed that study participation increased their overall wellbeing due to “giving to others.”*  *51SC “Giving something back*  *It was reported that participants’ decisions to take part in the MAVIS trial and trials in general included the consideration of being able to “giving something back” to the health service by taking part in the trial (n=18):*  *51PC “Yes. NHS has served me well over the years. Taking part in studies is my way of saying thank you.”*  *“Yes, to put something back into the health service.”*  *“Yes, if the studies appear to be worthwhile. A long time ago I did some research in a very different field and liked to be associated with research again even if the connection was very minor.”* | |
| Personal Benefits (20) | Personal gains for participants themselves from trial involvement | |
| - Regain a sense of control(1) | *3BPC “I just completed the forms as requested and it was no hassle at all. And actually it helped because it was something positive to do, on certain days and ticking the boxes and all that sort of thing, I felt because I think part of having cancer is you lose control, and I am quite, the sort of person that likes to be in control and this is enabling me a little bit of control back, so that part I quite enjoyed actually.”*  *3BSC “Specifically, that it provided them with some control, at a time when most felt a lack of control over their cancer experience;”* | |
| - Improve self discipline(1) | *25SC “Also, being enrolled in trials helped several participants to maintain self-discipline, crucial for people with chronic diseases who need to take drugs continuously”*  *25PC “‘I suffer from [hypertension]. And in such case, if you have high blood pressure, you should take the pill, if you don’t have high blood pressure you should still take the pill. Research disciplines me, so being enrolled in clinical studies played some sort of a positive role, I got used to do this. Some people forget, but I am used to this: wake up in the morning – take the pill, before going to bed – take the pill again’.* | |
| - Less responsibility and workload (1) | *6SC “In order to achieve the trial’s targets, UKPDS staff had to adopt a directive approach with patients. All patients indicated that they had appreciated this approach:”*  *6PC “I’m not criticising doctors, the local GPs or anything like that, because they haven’t got time to devote purely to diabetes. But the problem is that it puts a lot more onus onto the individual, i.e. myself, to control it much better, do the checks and everything else.”*  *6SC/PC “Several of them drew attention to the complex nature of the disease management process. This, combined with their fears of developing complications, had led them to value the input of UKPDS professionals who could ‘do the thinking, planning and worrying for [them]”* | |
| - Gain research knowledge through participation(2) | *2SC “All former trial participants said they felt more knowledgeable about trials and research since participating in a clinical trial.”*  *37SC “Despite the negative and potentially distressing ﬁndings of the Oracle Children’s Study, the majority of women still valued receiving the results. It enabled a sense of closure and completeness, and signiﬁed to women that the researchers were acknowledging their contribution.”*  *37PC “It was like you’d not been forgotten about really, that all of us that had done this, we’d done it and it had been acknowledged by getting the results.”* | |
| - Increased awareness of health status and psychological status(8) | *1PC “No trial ever has a negative, negativity. It always has a positive; there’s always something positive that comes out of it, even if it is only to say, ‘We don’t want to go up that route”*  *4SC “Participants felt that completing the questionnaire regarding our primary outcomes provided them with a regular self-assessment and valuable insights into their condition and emotions.”*  *4PC “They (the questionnaires) make me think, ‘How have I felt? How am I feeling?’ Because I have a tendency, maybe all people who are sick do, to kind of just cruise along saying, ‘I'm fine’.”*  *4PC “It gave me food for thought, it gave me more insight into perhaps what was really bothering me...some of the questions would bring to light, maybe some of the things I had been feeling, but didn't realize it until I had to answer.”*  *4PC “It also made me aware of any little changes....the answer might be, well, maybe a little different this time, or changed, which made me more aware of myself.”*  *6SC “The opportunity to receive regular clinical monitoring had provided the peace of mind of knowing that they were healthy or, if a secondary complication had emerged, that it would have been identified and acted upon immediately:”*  *6PC “And you had all these different tests and things done on your heart and everything, and you knew that you were, you know, everything was alright sort of thing.”*  *6PC “When I was on the study, I knew I was healthy. If something was wrong, I’m sure the X-rays and everything would have picked it up, if something was up.”*  *6PC “Also, you’re — how can I put it? You felt happy because the tests that you’ve had made you feel that you wouldn’t have had them if you didn’t come to the centre. You wouldn’t have had an MOT every three or four months. It does give you a lovely cushion to know that, whatever, they are going to pick something up, even if it’s not diabetic related, which I found very good”*  *19SC “Particularly for group 1 participants, reflecting on the benefits of participating in HSCT had brought involved a sense of gratitude at being given the opportunity to participate in the trial and being able to fulfil their ‘need to know’*  *25SC “Some participants also noted that through their involvement in clinical trials, they got to know their body better and learned to listen to it, which was helpful in managing their health conditions.”*  *25SC “They explained that this was mainly due to the requirement to monitor how one was feeling after taking the experimental drug and the need to report all changes and also due to their worries and curiosity about how the new substance would affect one’s body.”*  *25SC “Second, when allowed by the trial protocol, participants were provided with the results of their personal tests and were explained what they meant. Participants highly valued the opportunity to receive such information.”*  *25SC “Second, when allowed by the trial protocol, participants were provided with the results of their personal tests and were explained what they meant. Participants highly valued the opportunity to receive such information.”*  *31SC/PC “Completing the data collection tools was seen as a form of mental exercise for some patients:*  *“Sitting down to be asked questions made me better, because were it not for those questions it would have taken me a lot of time to get back to my normal psychological status.”*  *In particular, participants allocated to the control arm reported how completing the outcome measures prompted self-care:*  *“To be honest when I ﬁrst came here and sat down with you for a discussion I felt different…the discussion we used to have made me feel at peace and I got rid of all the bad thoughts I had, on my way home…so I started helping myself to change step by step.”*  *Other participants described how the PROMs provided an opportunity to consider making positive changes in their lives:*  *“When I used to come here, we would talk with you and you would ask me the questions and I’d reﬂect back… So I would think about it – reﬂect, reﬂect about it …and once I’d reﬂected I’d sit down and think, now, something should change.”*  *35SC “Patients also thought that completion of the questionnaires provided an alternative way to express, and thereby better understand, their pain and outcomes. The daily 100 mm pain visual analogue scale (VAS) pain scores that patients were asked to complete on days 1–3 postoperative while in hospital helped quantify the patients’ pain experience and learn more about their recovery from surgery.”*  *47SC “For one participant, completing a cancer-specific symptom checklist was useful in helping decrease her anxiety by normalizing symptoms.”* | |
| - Experience improved healthcare relationships(12) |  | |
| - - Reinforced trust(4) | *6SC “They described how the clinics provided a safe space in which to share concerns with professionals who had time to listen, and in whom they had developed trust over many years.”*  *31SC “Compassion, social support and communication related to development of positive and trusting relationships with the research team:”*  *46PC “Yes, basically I have trust in my doctor. I feel in good hands. (…) And of course, they now have a leap of faith. No, therefore I had no concerns that they would try anything or that something bad would happen to me.”*  *53SC “Trust in the clinical trials team. Trust in the clinical trials team was expressed in terms of the health professional being an expert, which extended to trust in the trial itself. The respondents were particularly moved by the “friendliness” of the trial staff, who were viewed as being empathic, and who made the respondents “feel at ease”*  *53PC “Trust in the clinical trials team*  *“When I first came here I was a bit nervous, you know. I’d never been in such a place but the doctors, like all the nurses, everybody, the staff was just excellent. They treat me really good and talked to me nicely...it was perfect.”* | |
| - - Receive support and encouragement(10) | *1SC “Some patients also perceived benefits from the additional contact trials gave with medical staff, as well as the personal qualities of these staff.”*  *1PC “They’ve all been interesting. I’ve met some lovely, interesting people…If they came to me for any other trials, I would partake.”*  *2PC “Dr. ____ said “we” whenever he talked about the cancer. It made me feel like I wasn’t alone. We were in this together.”*  *13SC “The trial and research activities provided opportunities to meet others who had been through similar experiences:”*  *23SC “the trial had given them hope and provided emotional support.”*  *24SC “They valued good interpersonal skills of the research staff, using words such as “friendliness”, “considerateness” and “competence” to describe the behaviour of research personnel who approached them. This seemed to generate trust and to promote participation.”*  *25SC “Such a ‘helpline’ was greatly appreciated by the participants.”*  *25PC “They are very nice, I can call any time and ask a question about any of my diseases, even unrelated to the research study.”*  *25SC “The vast majority of the respondents reported that an opportunity to support the long-term relationships with the investigators who were responsive to their needs and had in-depth knowledge of their medical histories was very valuable for them.*  *25SC “Furthermore, the respondents having a long experience in trial participation reported that their connection with the investigators did not break after the trial completed. The investigators still responded to their requests and offered to continue participation if a new suitable trial was planned at their site”*  *31SC “The relationship with the study team thus appears to have enabled many patients to rebuild their self-image, improving their mental health and well-being:”*  *31SC “Compassion, social support and communication related to development of positive and trusting relationships with the research team:”*  *31PC “For instance one may come here broken hearted and feeling down but you would encourage and give him the best.”*  *31SC “Participants derived social support from their peers in the study, from the study team and from religious practice, which the intervention nurses encouraged:”*  *47SC “additional benefits that were experienced through the social interaction with the study's health professionals”*  *47SC/PC “For another participant, the contact was important as it made her aware that there were other ladies going through similar experiences. Finally, for one lady, the contact helped with the void after treatment, with the regular contact being described powerfully as a ‘lifeline.”*  *47SC/PC While the role of the professionals and the relationship between participants was never directly enquired about, it emerged as a key theme when evaluating the programme. It appears that contact with the therapist established a relationship that was important to the success of the programme. This relationship, it appears, created an extra motivation to exercise.*  *[P2: More as a friend, wasn't she? (referring to the therapist) P1: Exactly. That's what my husband said. It's like your friend phoning you today or whatever, you know. That was it. That was how she was perceived, you know. P2: It wasn't just somebody doing their job. P1: No. P2: I think that's what helped too.]*  *[P11: And you build up a relationship with Caroline (therapist) you know P12: You do P10: You do.]”*  *51PC “I liked the friendly approach of those members of the team with which I came into contact with”.*  *“I liked the regular communication between researchers and subject. This gave me a good feeling about the quality of the study.”*  *“You kept in touch and I felt part of the programme.”*  *“Over the period in question, I was treated with kindness and helped throughout.”*  *53PC “When I first came here I was a bit nervous, you know. I’d never been in such a place but the doctors, like all the nurses, everybody, the staff was just excellent. They treat me really good and talked to me nicely...it was perfect.”* | |
| - - Shares worries and burdens(3) | *6SC “They described how the clinics provided a safe space in which to share concerns with professionals who had time to listen, and in whom they had developed trust over many years.”*  *6PC “This experience was particularly valued by those who felt unable to share their worries with their families and/or did not wish to burden their seemingly overstretched GPs:”*  *25SC “At the same time, the participants could call and ask for advice or share concerns not only with regard to the condition studied in a particular clinical trial but also in relation to other health issues they might have”*  *47SC “For some ladies, the contact with a health professional provided an avenue to discuss feelings or symptoms that they preferred not to burden or constantly burden family members or friends with.”*  *47PC “No, I found it really therapeutic. You know, I mean you're diagnosed with cancer, you have to accept OK we're going down this road now, you're going to hospital, getting all your treatment and then for being in this study this was another thing, I just viewed it as another thing that is going to help me through it and for getting phone calls, it was just fantastic. It was another avenue for me to offload all these feelings because my poor family had listened to so much and you felt nearly guilty, you know, saying the same thing over and over and over and over, again, and this was another person that I could say it to, you know, who wasn't emotionally involved and you get all of this off your chest and then say bye bye and that's was OK so…”* | |
| - - Felt at home and treated like family(3) | *1PC “It’s laidback; it’s, it’s like home from home. It’s, there’s no ‘oh you’re the patient, we’re the experts’.”*  *6SC”Several patients likened the UKPDS clinics to a ‘safe haven’ or a pseudo-surrogate family.”*  *6PC “Because they took time … they spent time with you. You felt like you were being looked after: you’re not a number, you’re a person. And sometimes you do feel that you’re perhaps just a number.”*  *31PC “It is because when you used to ask me questions, I used to feel much free inside … I felt like a very normal person without any form of illness, I felt so good. Female, 54 years, intervention”* | |
| - Receive special attention (10) |  | |
| - - Closer supervision of illness (5) | *1PC “Well, I think being part of the trial, you’re looked at better than if I wasn’t on the trial. You know, you’re being watched more, you know, and so, and because you see the research nurse. Otherwise, you are living on your own and you never see anyone. At least they are keep[ing] tabs on you.”*  *6SC “Patients spoke of their experiences of receiving regular, comprehensive clinical tests in uniformly positive ways. Far from being seen as an intrusive, albeit necessary part of trial participation, patients regarded the clinical examinations as constituting a major benefit”*  *6SC “Many patients also believed that they received a more personal form of care as a consequence of trial participation”*  *11PC “I will be very closely monitored throughout.” “The numerous medical check-ups which I would not normally have.”*  *25SC “First, they appreciated the regular checkups in the course of the clinical trial.*  *25SC “Being people with chronic health conditions, they felt reassured that their health state was under control due to the regular monitoring.”*  *25SC “At the same time, the participants could call and ask for advice or share concerns not only with regard to the condition studied in a particular clinical trial but also in relation to other health issues they might have”*  *25SC “Such a ‘helpline’ was greatly appreciated by the participants.”*  *C SC/PC “Participants reported three main reasons for joining the ZICE trial: the possibility of receiving the oral treatment, being constantly monitored and helping others. Some participants felt that participating in the trial, on either arm, would give them more security, as they were coming to the clinic more regularly and could therefore receive additional attention from the health-care professionals:*  *For me it means that there’s always somebody checking up on me more, so I feel a bit more secure. (R11, rural area, IV treatment)”* | |
| - - Closer contact with medical staff (6) | *1SC “Some patients also perceived benefits from the additional contact trials gave with medical staff, as well as the personal qualities of these staff.”*  *3BPC “Yes he was very supportive [hospital consultant] and he did ask me every time I went for an appointment how it was going.”*  *25PC “They are very nice, I can call any time and ask a question about any of my diseases, even unrelated to the research study.”*  *25SC “Furthermore, the respondents having a long experience in trial participation reported that their connection with the investigators did not break after the trial completed. The investigators still responded to their requests and offered to continue participation if a new suitable trial was planned at their site”*  *25PC “Now the trial I participated in is finished. But they say that in September a new trial will start. I told that I would like to participate. I like that in any moment, if I need anything, medical help will be provided to me. I am very satisfied with this”*  *31SC “Compassion, social support and communication related to development of positive and trusting relationships with the research team:*  *51PC “I liked the friendly approach of those members of the team with which I came into contact with”.*  *“I liked the regular communication between researchers and subject. This gave me a good feeling about the quality of the study.”*  *“You kept in touch and I felt part of the programme.”*  *“Over the period in question, I was treated with kindness and helped throughout.”*  *C SC/PC “Participants reported three main reasons for joining the ZICE trial: the possibility of receiving the oral treatment, being constantly monitored and helping others. Some participants felt that participating in the trial, on either arm, would give them more security, as they were coming to the clinic more regularly and could therefore receive additional attention from the health-care professionals:*  *For me it means that there’s always somebody checking up on me more, so I feel a bit more secure. (R11, rural area, IV treatment)”* | |
| - - Extra efforts made especially for trial participants (2) | *24SC “A certain degree of convenience in pre-arranging reception processes and ideally providing patients the possibility to get the blood test done locally to reduce time required were mentioned as important aspects to make it as convenient as possible,”*  *24PC “Actually he really made it very smooth. They did a little note that I could bring with me in the evening and he had arranged for the A&E reception to have my details so that when I turned up I could literally just go in, have the blood test taken and go home so to make that quite easy.”*  *24SC “Interviewees did express that participation in the trial provided the benefit of getting some “extra care” including attention and better follow up due to participating in the trial.”*  *51PC “Over the period in question, I was treated with kindness and helped throughout.”*  *51PC ““I thought it was very well organised. The research nurses were very friendly and it was specially kind of them to send out cards to us at Christmas.”* | |
| - - Superior healthcare quality, facilities and personnel (4) | *2PC “felt privileged to be on a trial. We had a separate chemo facility and we all knew we were at the Ritz.”*  *11SC “Another less common personal benefit was that participants will have access to high-quality medical care, which is probably the case in most oncology clinical trials in South Africa”*  *6SC “Consequently, patients welcomed the opportunity to join the UKPDS because they believed this would enable them to receive ‘the most up-todate, state-of-the-art care’ (Male 5) from professionals with a specialist expertise in diabetes:”*  *6PC “Everybody, I think, who decided to come here, recognised how well up they were on the programmes at the time, you know. They seemed to know a lot, so I was happy to be under them.”*  *6SC “In their opinion, this was made possible by high levels of staff continuity, and by the fact that UKPDS staff did not appear to have to operate within the same tight time constraints as GPs and other medical practitioners.”*  *24SC “Interviewees did express that participation in the trial provided the benefit of getting some “extra care” including attention and better follow up due to participating in the trial.”*  *24SC “They valued good interpersonal skills of the research staff, using words such as “friendliness”, “considerateness” and “competence” to describe the behaviour of research personnel who approached them. This seemed to generate trust and to promote participation.”* | |
| - Means to be gainfully occupied (4) | *1SC “Participating in research also provided him with a role, with activities to pass the time and help alleviate the boredom, which he experienced after having to give up work when he became ill. He found being a research participant ‘fun’ and appreciated the attention from research staff.”*  *1PC “Yeah, yeah, I mean, it’s something to do, you know; it’s good fun, it breaks things up. Life gets a bit boring when you are stuck like this, you know.”*  *24PC “The participants were also motivated by a sense that the research was worthwhile.”*  *35SC “Many stated that trial participation ‘gave them something to do’ while recovering from surgery and that they had sufﬁcient time available to complete questionnaires.”*  *APC “Participating gives me a sense of usefulness. It makes the disease less useless.’ (ALS)* | |
| - Monetary Incentives (2) | *31SC “Participants used the money they received for participating in the study to buy food for themselves and their dependents, enabling participants to fulﬁl their social role as providers for their families:”*  *31PC “That fare, it really amazed me. It gave me special joy such that whenever you told me to come here. I’d feel delighted and say, “Today we must eat some chicken – I’m a going to be rich”. So this made me very happy.”*  *46PC “Firstly the drugs were free, which I found good. And you did not have to pay five Euros [pharmacy charge], which was also a factor.”* | |

1. **Table of primary full-text reports included in systematic review and thematic synthesis**

| **Title** | **Year** | **Clinical Domain** | **Number of Total Participants in the Qualitative Study** | **Data Collection Methods** | **Data Analysis Methods** |
| --- | --- | --- | --- | --- | --- |
| “I didn’t really understand it, I just thought it’d help”: exploring the motivations, understandings and experiences of patients with advanced lung cancer participating in a non-placebo clinical IMP trial | 2016 | Cancer | 10 | Interviews | Interpretative Phenomenological Analysis |
| The Guinea Pig Syndrome: Improving Clinical Trial Participation among Thoracic Patients | 2007 | Cancer | 18 | Interviews | Content analysis |
| “Until the trial is complete you can’t really say whether it helped you or not, can you?”: exploring cancer patients’ perceptions of taking part in a trial of acupressure wristbands | 2013 | Cancer | 26 | Interviews | Thematic Analysis |
| Patient Perspectives on Participation in the ENABLE II  Randomized Controlled Trial of a Concurrent Oncology Palliative Care Intervention: Benefits and Burdens | 2013 | Cancer | 53 | Interviews | Thematic Analysis |
| Participating in the United Kingdom Prospective Diabetes Study (UKPDS): a qualitative study of patients’ experiences | 2003 | Chronic diseases | 10 | Interviews | Grounded Theory |
| Identifying components in consent information needed to support informed decision making about trial participation: An interview study with women managing cancer | 2016 | Cancer | 15 | Interviews | Thematic Analysis |
| Participation in clinical research: perspectives of adult patients and parents of pediatric patients undergoing hematopoietic stem cell transplantation | 2014 | Cancer | 17 | Focus groups | Content analysis |
| Attitudes towards clinical research among cancer trial participants and non-participants: an interview study using a Grounded Theory approach | 2006 | Cancer | 14 | Interviews | Grounded Theory |
| Phase 3 Oncology Clinical Trials in South Africa: Experimentation or Therapeutic Misconception? | 2016 | Cancer | 29 | Open-Ended Survey Questions | Content analysis |
| Perceptions of equipoise are crucial to trial participation: a qualitative study of men in the ProtecT study | 2003 | Cancer | 21 | Interviews | Thematic Analysis |
| Receiving or not receiving acupuncture in a trial: The experience of participants recovering from breast cancer treatment | 2014 | Cancer | 40 | Focus groups | Thematic Analysis |
| Scientific tools, fake treatments, or triggers for psychological healing: How clinical trial participants conceptualise placebos | 2012 | Chronic diseases | 12 | Interviews | Thematic Analysis |
| Living with Crohn’s disease: an exploratory cross-sectional qualitative study into decision-making and expectations in relation to autologous haematopoietic stem cell treatment (the DECIDES study) | 2017 | Chronic diseases | 22 | Interviews | Thematic Analysis |
| Patients’ perceptions of information provided in clinical  trials | 2001 | Mixed | 78 | Interviews | Not Reported |
| Challenges of maintaining research protocol fidelity in a clinical care setting: A qualitative study of the experiences and views of patients and staff participating in a randomized controlled trial | 2011 | Chronic diseases | 45 | Interviews | Grounded Theory |
| Close look at the experiences of patients enrolled in a clinical trial of acupuncture treatment for atrial fibrillation in Korea: a qualitative study nested within a randomised controlled trial | 2017 | Chronic diseases | 8 | Interviews | Thematic Analysis |
| An exploration of patients’ experiences of participation in a randomised controlled trial of the Manchester Acute Coronary Syndromes (MACS) decision rule | 2017 | Acute Illness | 10 | Interviews | Thematic Analysis |
| Risks and benefits of trial participation: A qualitative study of participants’ perspectives in Russia | 2015 | Chronic diseases | 21 | Interviews | Grounded Theory |
| How experiences become data: the process of eliciting adverse event, medical history and concomitant medication reports in antimalarial and antiretroviral interaction trials | 2013 | Acute Illness | 27 | Interviews | Thematic Analysis |
| Participants’ perceptions and understanding of a malaria clinical trial in Bangladesh | 2014 | Acute Illness | 16 | Interviews | Thematic Analysis |
| Conducting experimental research in marginalised populations: clinical and methodological implications from a mixed-methods randomised controlled trial in Kenya | 2016 | Chronic Diseases | 30 | Interviews | Thematic Analysis |
| Internet-based trials and the creation of health consumers | 2010 | Mental health | 10 | Interviews | Thematic Analysis |
| Response and non-response to postal questionnaire follow-up in a clinical trial – a qualitative study of the patient’s perspective | 2006 | Orthopedics | 22 | Interviews | Thematic Analysis |
| Understanding involvement in surgical orthopedic randomized controlled trials: A qualitative study of patient and health professional views and experiences | 2016 | Orthopedics | 24 | Interviews | Thematic Analysis |
| Testing a drug during labour: the experiences of women who participated in a clinical trial | 2000 | Obstetrics | 26 | Interviews | Not reported |
| Consent revisited: the impact of return of results on participants’ views and expectations about trial participation | 2015 | Obstetrics | 38 | Interviews | Grounded Theory |
| Deciding to join a perinatal randomised controlled trial: Experiences and views of pregnant women enroled in the Magpie Trial | 2011 | Obstetrics | 40 | Interviews | Thematic Analysis |
| Participating in a trial in a critical situation: a qualitative study in pregnancy | 2006 | Obstetrics | 20 | Interviews | Grounded Theory |
| Participation in a randomised controlled trial of acupuncture as an adjunct to in vitro fertilisation: the views of study patients and acupuncturists | 2015 | Gynecology | 146 | Interviews | Thematic Analysis |
| Pregnant womens’ concerns when invited to a randomized trial: a qualitative case control study | 2015 | Obstetrics | 12 | Interviews | Grounded Theory |
| Recruiting and consenting into a peripartum trial in an emergency setting: a qualitative study of the experiences and views of women and healthcare professionals | 2016 | Obstetrics | 22 | Interviews | Grounded Theory |
| Unblinding following trial participation: Qualitative study of participants’ perspectives | 2013 | Obstetrics | 38 | Interviews | Grounded Theory |
| Trial participation as avoidance strategy: a qualitative study | 2015 | Uro-gynecology | 29 | Interviews | Grounded Theory |
| Why do – or don’t – patients with urinary tract infection participate in a clinical trial? A qualitative study in German family medicine | 2015 | Acute Illness | 20 | Interviews | Content analysis |
| A focus group study exploring gynecological cancer survivors’experiences and perceptions of participating in a RCT testing the efficacy of a home-based physical activity intervention | 2013 | Cancer | 16 | Focus groups | Thematic Analysis |
| Cancer Clinical Trial Patients in the Information Age | 2001 | Cancer | 42 | Open-Ended Survey Questions | Content analysis |
| Experiences of randomization: Interviews with patients and clinicians in the SPCG-IV trial | 2008 | Cancer | 5 | Interviews | Content analysis |
| Women’s views and experiences of a patient preference trial in surgery: a qualitative study of the CARPET1 trial | 2010 | Uro-gynecology | 16 | Interviews | Grounded Theory |
| Factors influencing the participation of older people in clinical trials – Data analysis from the Mavis trial | 2010 | Chronic diseases | 540 | Open-Ended Survey Questions | Content analysis |
| “Playing Their Parts”: The Experiences of Participants in a  Randomized Sham-Controlled Acupuncture Trial | 2008 | Chronic diseases | 10 | Interviews | Thematic Analysis |
| South Asian patients’ views and experiences of clinical trial participation | 2004 | Mixed | 15 | Interviews | Thematic Analysis |
| Public awareness of clinical trials: A qualitative pilot study in Pune | 2012 | Mixed | 7 | Interviews | Content analysis |
| Participating in clinical trials: participants’ experiences. A qualitative study |  | Chronic diseases | 50 | Interviews | Thematic Analysis |
| QUALZICE: A QUALitative exploration of the experiences of the participants from the ZICE clinical trial (metastatic breast cancer) receiving intravenous or oral bisphosphonates | 2013 | Cancer | 42 | Interviews | Interpretative Phenomenological Analysis |
| Clinical trial participants’ experiences of completing questionnaires: a qualitative study | 2014 | Chronic diseases | 20 | Interviews | Content analysis |
